# A Streamlined Method to Obtain Biologically Active TcdA and TcdB Toxins from *Clostridioides difficile*

**DOI:** 10.3390/toxins16010038

**Published:** 2024-01-11

**Authors:** Diane Sapa, Anaïs Brosse, Héloïse Coullon, Gauthier Péan de Ponfilly, Thomas Candela, Alban Le Monnier

**Affiliations:** 1 Micalis Institute, Université Paris-Saclay, INRAE, AgroParisTech, 78350 Jouy-en-Josas, France; afi-akofa-diane.sapa@universite-paris-saclay.fr (D.S.); heloise.coullon@universite-paris-saclay.fr (H.C.); gpeandeponfilly@ghpsj.fr (G.P.d.P.); thomas.candela@universite-paris-saclay.fr (T.C.); alban.le-monnier@universite-paris-saclay.fr (A.L.M.); 2 Service de Microbiologie Clinique, GH Paris Saint-Joseph, 75674 Paris, France

**Keywords:** *Clostridioides difficile*, toxin, recombinant protein, cytotoxic activity, TcdA, TcdB

## Abstract

The major virulence factors of *Clostridioides difficile* (*C. difficile*) are enterotoxins A (TcdA) and B (TcdB). The study of toxins is a crucial step in exploring the virulence of this pathogen. Currently, the toxin purification process is either laborious and time-consuming in *C. difficile* or performed in heterologous hosts. Therefore, we propose a streamlined method to obtain functional toxins in *C. difficile*. Two *C. difficile* strains were generated, each harboring a sequence encoding a His-tag at the 3′ end of *C. difficile* 630∆*erm tcdA* or *tcdB* genes. Each toxin gene is expressed using the P_tet_ promoter, which is inducible by anhydro-tetracycline. The obtained purification yields were 0.28 mg and 0.1 mg per liter for rTcdA and rTcdB, respectively. In this study, we successfully developed a simple routine method that allows the production and purification of biologically active rTcdA and rTcdB toxins with similar activities compared to native toxins.

## 1. Introduction

*Clostridium difficile* (*C. difficile*) is a Gram-positive, anaerobic, spore-forming, toxin-producing bacillus that was renamed *Clostridioides difficile* in 2016 [1]. *C. difficile* was initially identified as part of the flora of healthy infants in 1935 [2]. It was subsequently described as a causative agent of digestive tract infections often involved in antibiotic-associated diarrhea ranging from mild to severe and life-threatening complications such as pseudomembranous colitis. *C. difficile* infections (CDI) are associated with high recurrence rates, reaching up to 30% of cases [3,4,5]. CDI has been recognized as a leading cause of healthcare-associated infections and, more generally, a substantial threat to public health [6,7,8]. The main risk factor for developing CDI is antibiotic therapy [9], which can disrupt the gut microbiota. In this context of dysbiosis, spores of *C. difficile* will germinate into vegetative forms, which will then colonize the gut microbiota, multiply, and produce virulence factors, in particular enterotoxins A (TcdA) and B (TcdB) [10], that lead to the development of symptoms. TcdA and TcdB are encoded by the *tcdA* and *tcdB* genes, localized in the 19.6 kb pathogenesis locus (PaLoc), along with three additional genes that allow regulation and release of the toxins (*tcdR*, *tcdC*, and *tcdE*, [11]). TcdA and TcdB are expressed when bacterial cells enter the stationary phase, likely caused by the limitation of nutrients [12]. TcdA and TcdB belong to the family of Large Clostridia Toxins (LCTs) due to their high molecular weight (308 and 270 kDa, respectively). The LCTs can induce profound changes in cell morphology [13]. These toxins are composed of four functional domains: the N-terminal glucosyl-transferase region, the auto-processing cysteine protease domain, the C-terminal membrane translocation region (CROPS), and Receptor-Binding Domain region. After binding to their receptors on the cell surface, endocytosis is initiated, and toxins are internalized into endosomes. Subsequent acidification of the endosomes induces a conformational change of the delivery domain, resulting in pore formation and the translocation of a catalytic glucosyltransferase domain across the endosomal membrane. The toxins subsequently undergo autocleavage, which releases the N-terminal glucosyltransferase region into the cytoplasm [14]. The glucosyltransferase activity domain targets and inactivates Rho GTPases, including Rac1 and Cdc42 at Thr35 as well as RhoA at Thr37. Inactivation of Rho GTPases leads to actin-depolymerization, which results in cell rounding, disaggregation of the actin cytoskeleton, loss of intestinal epithelial barrier function, and cell death. In addition to their direct cytotoxic effects, TcdA and TcdB elicit a pro-inflammatory response, contributing to tissue damage and leading to severe complications such as pseudomembranous colitis [15,16]. In recent years, studies have provided further insight into the structure of TcdA and TcdB across *C. difficile* strains, leading to the identification of distinct subtypes of toxins [17]. Of note, clade 2 hypervirulent *C. difficile* strains have been linked to the expression of toxin subtypes TcdB2 and TcdB4. Importantly, subtypes of TcdB toxins have also been linked to the recognition of distinct receptors within the host [18,19]. Taken together, these recent results highlight the need for adequate tools for accurate toxin studies.

Having biologically functional purified toxins is an important tool to understand these toxins’ activities in the modulation of the pathogenesis of CDI, the host immune response, and, more importantly, for the development of CDI treatments. In addition, clinical research relies on *C. difficile* toxins to quantify anti-TcdA or TcdB antibodies by ELISA or for neutralizing antibody tests. The native toxins are usually purified from toxigenic *C. difficile* VPI 10463 culture supernatant [20,21,22], but the purification process is fastidious, time-consuming, and requires multiple steps. In this study, we aimed to develop a simplified method for the purification process of biologically active native *C. difficile* TcdA and TcdB by directly using *C. difficile* toxigenic strain 630∆*erm* as the host.

## 2. Results

### 2.1. Selection of a C. difficile Strain

**Figure 1:**
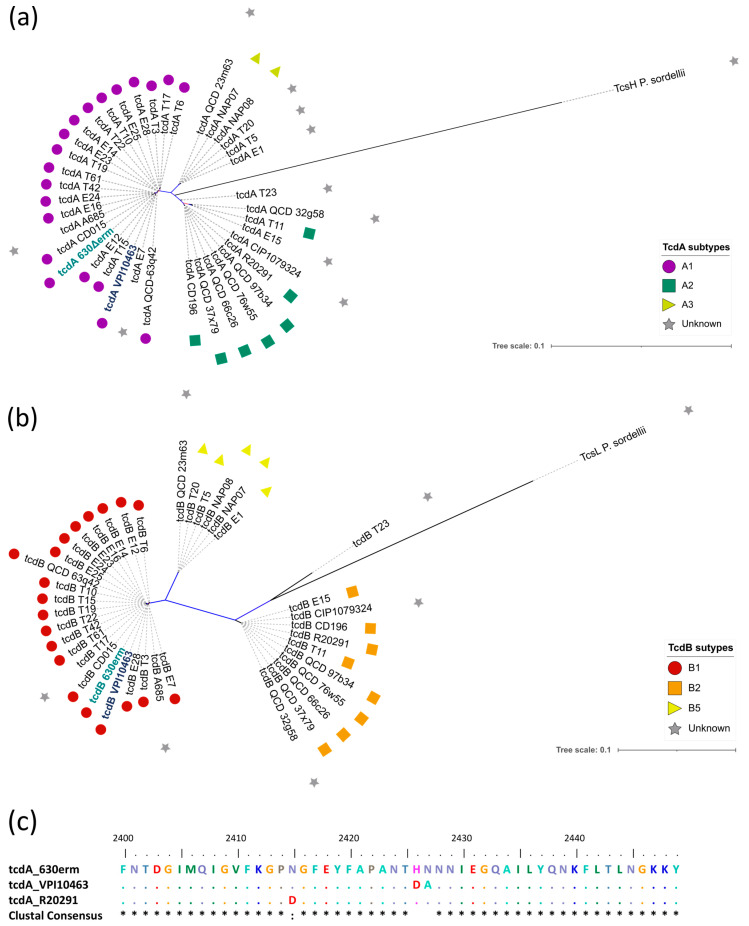
Toxins of the 630Δ*erm* and VPI 10463 strains are found in shared clusters. Unrooted phylogenetic trees of (**a**) TcdA and (**b**) TcdB proteins obtained from 39 *C. difficile* genomes, with bootstrap values represented as branch colors ranging from 0 in red to 1 in blue. Symbols represent the toxin subgroups for each protein in the trees: TcdA1 = purple circle/TcdA2 = green square/TcdA3 = yellow triangle/TcdB1 = red circle/TcdB2 = orange square/TcdB5 = yellow triangle. In both graphs, gray stars represent proteins for which the toxin subtype is either unknown (*C. difficile* proteins) or not applicable (TcsH and TcsL). Reference sequences from the *C. difficile* 630Δ*erm* and VPI 10463 strains are indicated in bold letters. In addition, each tree contains the closest Large Clostridial Toxin for the respective toxin (TcsH from *P. sordellii* for TcdA/TcsL from *P. sordellii* for TcdB). (**c**) Multiple sequence alignment of positions 2400 to 2450 of TcdA proteins using *C. difficile* 630Δ*erm*, VPI 10463, and R20291 strains, with the resulting clustal consensus sequence. Identical amino acids are represented as dots.

Since previous studies relied on the VPI 10463 strain for the purification of TcdA and TcdB, we first sought to determine whether the native toxins from the 630Δ*erm* strain were structurally comparable to the toxins from the VPI 10463 strain. We performed a protein sequence comparison for TcdA and TcdB across 39 *C. difficile* strains and generated phylogenetic trees of each protein list (Figure 1, panels a and b). Each protein entry was subsequently annotated based on their described toxin subtypes [17]. Analysis of these trees showed a distribution of TcdA proteins in tree-distinct clusters, which appeared consistent with the toxin subtypes (Figure 1a; TcdA1, TcdA2, TcdA3). Similarly, TcdB proteins appeared in three distinct clusters, consistent with three TcdB subtypes (Figure 1b; TcdB1, TcdB2, and TcdB5). In these trees, the TcdA and TcdB proteins of the 630Δ*erm* and VPI 10463 strains were found in the same clusters, suggesting that they are of the same toxin subtype (TcdA1 and TcdB1, respectively). In addition, percent identity matrixes showed identity scores of 99.82% and 100% for TcdA and TcdB, respectively (File S1). Of note, the full-length alignment of TcdA_630Δ*erm*_ and TcdA_VPI 10463_ showed only two differing residues between both proteins at position 2421–2422, which corresponds to the CROPS domain associated with membrane translocation (Figure 1c and File S1). Taken together, these results demonstrate that the toxins of the 630Δ*erm* are nearly identical to those of the VPI 10463 strain, confirming the potential use of the 630Δ*erm* as a replacement for the VPI 10463 strain for toxin production.

### 2.2. Expression and Purification of Recombinant Toxins

We then constructed two pseudo-suicide plasmids, pADS1 and pADS2. These plasmids were used to transfer a sequence encoding a His-tag at the 3′ end of *tcdA* and *tcdB* genes by conjugation and generate *C. difficile* strains AD1 and AD2 (Figure S1), respectively, for the production of recombinant toxins rTcdA and rTcdB. The resulting AD1 (*tcdA*-His) and AD2 (*tcdB*-His) strains were grown in TY medium for 75 to 92 h to induce *tcdA* and *tcdB* expression under physiological conditions. rTcdA and rTcdB were then purified by Ni-NTA chromatography affinity under denaturing conditions. The rTcdA and rTcdB proteins were not found in the raw protein fraction, the flowthrough, or the eluted fractions (Figure S2). These results suggest that we could not purify rTcdA and rTcdB using these culture conditions, probably due to a lack of *tcdA* and *tcdB* expression.

**Figure 2:**
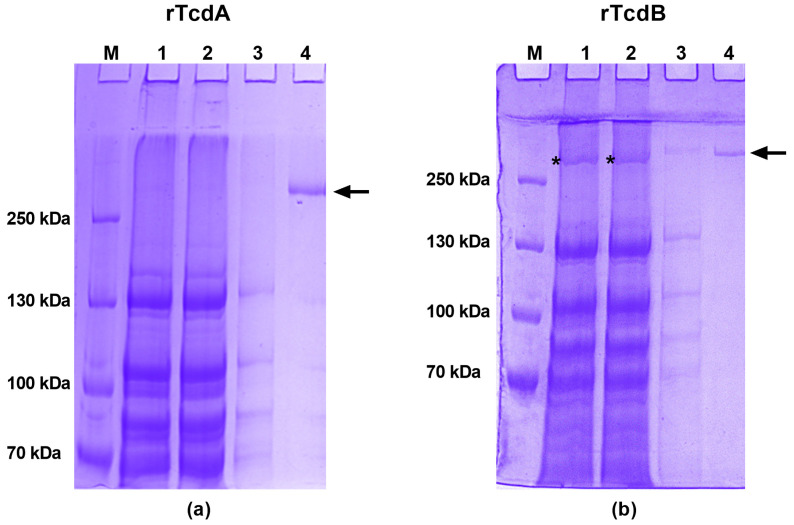
Purification of recombinant toxins by Ni-NTA affinity chromatography under native conditions. Toxins were visualized by Coomassie staining for (**a**) recombinant toxin A (rTcdA) and (**b**) recombinant toxin B (rTcdB). M: PageRuler Plus marker (ThermoFisher)/1: total bacterial lysate/2: flow through/3: 10 mM imidazole wash/4: eluates after PD10. The SDS-PAGE gels shown are representative of at least 10 purifications. Arrows indicate the location of rTcdA and rTcdB, respectively. The asterisk indicates *C. difficile* proteins unrelated to rTcdB.

To control and optimize the production yield of *C. difficile* toxins, we replaced *C. difficile tcdA* and *tcdB* gene promoters with the ATc-inducible P_tet_ promoter in AD1 and AD2, respectively. This gave rise to *C. difficile* strains AD3 and AD4, which contain the promoter P_tet_ in front of the *tcdA* and *tcdB* open reading frames and a sequence encoding a His-tag in the 3′ end of each gene (Figure S1). The recombinant AD3 (P_tet_-*tcdA*-His) and AD4 (P_tet_-*tcdB*-His) strains were used to purify rTcdA and rTcdB, respectively, after 4 h of induction by ATc using Ni-NTA affinity chromatography under native conditions. This purification was carried out with the cell pellets rather than the supernatant. Indeed, as shown in Figure S3, no proteins with a size compatible with toxins were detected from the culture supernatant (Figure S3a,b). After purification, SDS-PAGE gels showed an expected band of about 308 kDa (Figure 2a, lane 4) corresponding to rTcdA and an expected band of about 270 kDa (Figure 2b, lane 4) corresponding to rTcdB. Moreover, the identity of the proteins identified in these bands was confirmed by Western blot (Figure S3, panel c and File S2 for experimental details). Of note, one band was detected in the total bacterial lysate as well as the flow through (Figure 2 panel (b) lanes 1 and 2). Since this protein was not retained in the Ni-NTA column, this indicates that it did not correspond to rTcdB but rather unrelated *C. difficile* proteins. DNA contamination was also estimated by Nanodrop measurement before and after treatment of rTcdA or rTcdB protein solutions by DNAse (Figure S3, panel d, and File S2 for experimental details). These results suggest that there might be a degree of DNA contamination, particularly for rTcdB. Altogether, rTcdA and rTcdB purification yields are 0.28 mg and 0.1 mg per liter of initial culture, respectively, as determined by the Bradford assay. Our new approach allows efficient and straightforward purification of the TcdA and TcdB toxins.

### 2.3. Recombinant Purified Toxins Possess Similar Biological Activity Compared to Native Toxins

**Figure 3:**
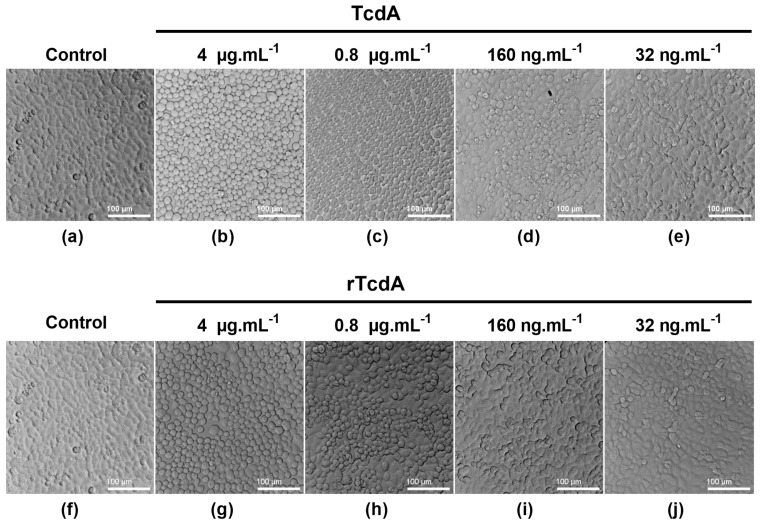
Cytotoxicity assay on Vero cells: TcdA versus rTcdA. Cells seeded in 96-well plates were incubated in the absence of toxin (**a**,**f**) or with decreasing concentrations (4 µg.mL^−1^, 0.8 µg.mL^−1^, 160 ng.mL^−1^, and 32 ng.mL^−1^) of either TcdA (**b**–**e**) or rTcdA (**g**–**j**). Cells were incubated for 18 h, and morphological changes were observed under phase contrast microscopy.

**Figure 4:**
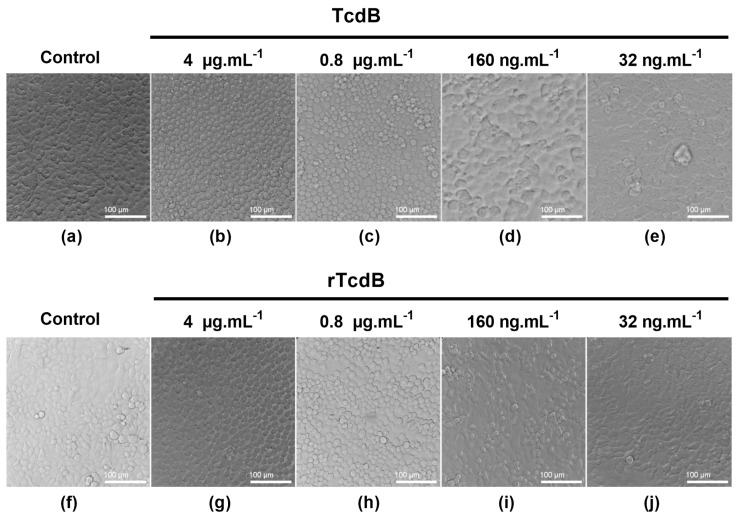
Cytotoxicity assay on Vero cells: TcdB versus rTcdB. Cells seeded in 96-well plates were incubated in the absence of toxin (**a**,**f**) or with decreasing concentrations (4 µg.mL^−1^, 0.8 µg.mL^−1^, 160 ng.mL^−1^, and 32 ng.mL^−1^) of either TcdB (**b**–**e**) or rTcdB (**g**–**j**). Cells were incubated for 18 h, and morphological changes were observed under phase contrast microscopy.

The functional activities of rTcdA and rTcdB were tested on Vero cells. First, we compared the cytotoxicity activity of the recombinant toxins with that of native toxins. After 18 h of incubation, cells incubated with either native toxin TcdA, TcdB, purified rTcdA, or purified rTcdB showed cell rounding at 4 µg.mL^−1^ (Figure 3b,g and Figure 4b,g, respectively), compared to the healthy confluent morphology observed for the negative controls (cells incubated without toxins—Figure 3a,f, and Figure 4a,f). Toxin activity decreased due to decreasing toxins concentrations, with no detectable cell rounding at 32 ng.mL^−1^ (Figure 3e,j and Figure 4e,j). These results suggest that rTcdA and rTcdB have similar cytotoxicity activity compared to native toxins on Vero cells and that this effect is dose-dependent.

**Figure 5:**
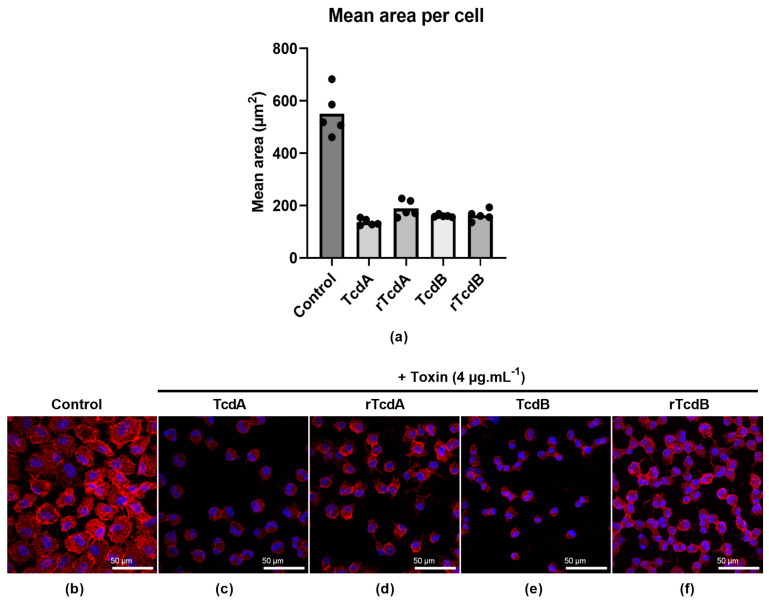
Disruption of the tight junctions on Vero cells. Cells in a 24-well plate were in the absence of toxin (control) or in the presence of 4 µg.mL^−1^ of either native or recombinant toxins A and B. Cells were incubated for 18 h, fixed, and stained with Rhodamine Phalloidin (actin staining in red) and DAPI (nuclei staining in blue), then observed under confocal microscopy with a 20× objective to quantify the effect of toxins on the cells. (**a**) Five random fields were taken to perform an analysis with FIJI imaging software version 2.9.0/1.53t. (**b**–**f**) Cell morphology changes were observed with 63× objective for (**b**) the control, (**c**,**e**) native toxins, and (**d**,**f**) purified recombinant toxins at 4 µg.mL^−1^.

We then explored if purified rTcdA and rTcdB could also induce actin cytoskeleton remodeling, as described for the native toxins. Incubation of cells with either native toxins or purified recombinant toxins leads to a decrease in cell size compared to the negative control (Figure 5a and Figure S4 for lower magnification). Furthermore, the decrease is similar between native and purified recombinant toxins. We then looked closely at the morphological changes of the cells (Figure 5b), and we found that the actin cytoskeleton was completely disrupted with both native (Figure 5c,e) and recombinant toxins (Figure 5d,f). Taken together, these results demonstrate that the purified recombinant toxins are as active as the native toxins.

### 2.4. Recombinant Toxins Can Be Used as Substitutes for Native Toxins in Clinical CDI Investigational Studies

**Figure 6:**
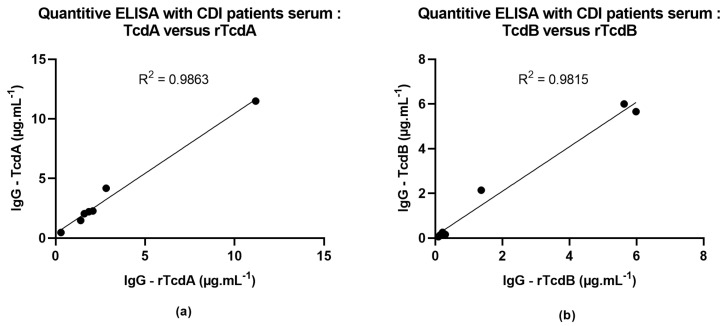
Comparison of native or recombinant toxins by quantitative ELISA. We determined the IgG concentrations in 7 samples of serum from CDI-recovered patients by quantitative ELISA for (**a**) TcdA or (**b**) TcdB. Graphs are plotted with the IgG titers obtained with recombinant or native toxins on the *x* and *y* axes, respectively. The R^2^ represents the Pearson correlation coefficient.

The native TcdA and TcdB toxins are currently used in several clinical research assays, including indirect ELISA assays and neutralization antibody assays. We first wanted to evaluate the use of purified recombinant toxins as coating antigens in quantitative indirect ELISA to measure anti-TcdA and TcdB antibody titers. Thus, we compared the concentration of IgG determined for several sera from CDI-recovered patients using assays done with native toxins and assays done with purified recombinant toxins. We obtained equivalent concentrations of IgG in both native and recombinant toxins assays, with a strong correlation as determined by the Pearson correlation coefficient (Figure 6a,b).

**Figure 7:**
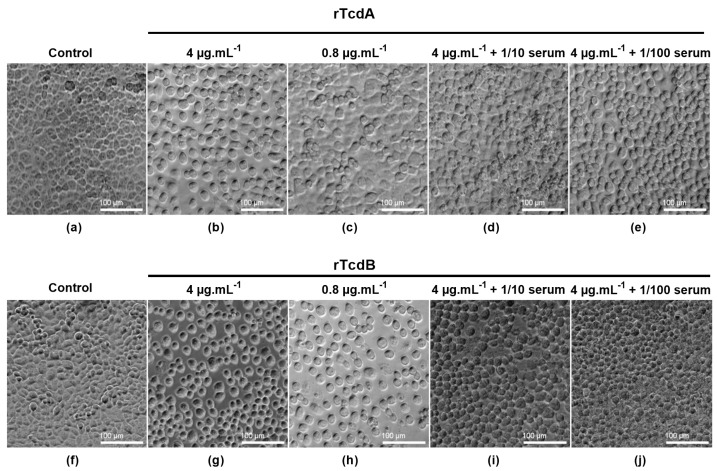
Neutralization assay of recombinant toxins on Vero cells. Cells in 96-well plates were incubated (**a**,**f**) in the absence or with (**b**–**e**) rTcdA and (**g**–**j**) rTcdB at 4 µg.mL^−1^, 0.8 µg.mL^−1^, and 75 µL of 4 µg.mL^−1^ of toxins preincubated with 75 µL of either 1/10 (**d**,**i**) or 1/100 (**e**,**j**) dilutions of CDI-recovered patient serum. Cells were incubated for 18 h, and morphological changes were observed under phase contrast microscopy.

Finally, we performed a neutralization antibody assay, comparing both sets of toxins. In these assays, we showed that incubation of the toxins with serum samples from CDI-recovered patients leads to a reduction in the cytotoxic effect of both rTcdA (Figure 7d,e) and rTcdB (Figure 7i,j). These results indicate that the effect of purified recombinant toxins can be neutralized by serum antibodies from a CDI-recovered patient, confirming that the C-terminal His-tag does not hinder detection of the protein itself. A comparison of the neutralization of native and recombinant toxins was investigated using a serum sample shown to contain neutralizing IgG against TcdB but none against TcdA (Figures S5 and S6). As expected, neither the native nor the recombinant TcdA could be neutralized by this serum (Figure S5), while both the native and recombinant TcdB were effectively neutralized up to 1/80 serum dilution (Figure S6). Taken together, these results suggest that the purified recombinant toxins are adequate substitutes for native toxins and their use in clinical research assays.

## 3. Discussion and Conclusions

*C. difficile* pathogenicity is linked to the production of two major virulence factors, the toxins A (TcdA) and B (TcdB). Multiple studies have increased our knowledge about the regulation, structures, and functions of *C. difficile* toxins. Recently, toxin subtypes have been identified and linked to various *C. difficile* strain clades, as well as recognition of different host receptors [17,18,19]. To provide new insights about their involvement in the pathophysiology of *C. difficile* infections, we need to have readily available biologically active toxins. Purified TcdA and TcdB have been useful research tools to access the host immune response against CDI by detecting the antibody titers by ELISA assays [23,24,25]. TcdA and TcdB have also been used in immunization assays for the production of anti-TcdA and TcdB antibodies required in immunoblotting. Their active forms have been used to study the cellular effects of these toxins in cytotoxicity assays [26], as well as in neutralization assays to study the neutralizing potential of the antibodies produced by CDI patients [27,28].

Previous purification methods of TcdA and TcdB in their native forms share similarities with those used for the purification protocols of toxins produced by other *Clostridium* spp. (*C. septicum*, *C. sordellii*, and *C. perfringens*) [20,29,30,31,32]. In contrast with our new purification method, the purification process of native toxins is laborious and time-consuming as it requires multiple steps, such as four to five days of growth in medium, ammonium sulfate precipitation, and ion exchange chromatography followed by gel filtration chromatography.

To simplify this process and obtain biologically active toxins, generating recombinant toxins in a heterologous host seemed to be a viable strategy to adopt. Over the years, in the case of *C. difficile* toxins, multiple attempts have been made to purify TcdA and TcdB in *E. coli* strains, an expression system mostly used to produce recombinant proteins. Because of the large size of the *tcdA* and *tcdB* genes, the expression of the entire toxin gene in *E. coli* is time-consuming and traditionally consists of the reconstruction of cloned fragments [33]. Moreover, purification of the full-length toxin in *E. coli* was only shown for TcdA. Other studies reported the purification of fragments of TcdA or TcdB [34]. The folding of such a toxin is therefore not guaranteed. Purifying toxins from the original host is more adequate to study their structure or potential post-translational modifications. In addition, *E. coli*, Burger et al. and Yang et al. have successfully expressed and purified biologically active recombinant TcdA and TcdB using *Bacillus megaterium* [35,36]. We asked for the recombinant *B. megaterium* strains expressing TcdA and TcdB. However, we could not obtain adequate titers of recombinant toxins due to difficulties in the lysis process. Finally, there are other alternatives to obtaining these toxins, such as commercial toxins, but they are expensive and do not guarantee biological activity.

Because of these limitations and our need to use active toxins in our studies, we aimed to develop a method that simplifies the purification process of *C. difficile* TcdA and TcdB by directly using *C. difficile* strain 630∆*erm* as the host. We obtained different yields for rTcdA and rTcDB, most likely due to the use of different ATc concentrations for the induction (0.5 µg.mL^−1^ and 0.1 µg.mL^−1^, respectively). We tried increasing the concentration of Atc for the induction of rTcdB but obtained lower yields. This could be a reflection of the higher cell toxicity of TcdB or the lower fitness of the mutant strain expressing rTcdB. For instance, higher expression of tcdB could lead to higher expression of genes downstream of *tcdB*, such as *tcdE*. Since TcdE acts as a holin-like protein [37], its overexpression could increase cell lysis, therefore reducing protein yield for rTcdB. However, this hypothesis was beyond the scope of our work and was therefore not investigated further. In the validation of the recombinant proteins purified, we could not exclude the presence of DNA contamination in the purified proteins. Given that bacterial DNA could be sensed by immune receptors of the host, such as TLR9 [38], researchers interested in using those proteins in immunology studies should consider adding extra steps to guarantee the removal of DNA contaminants. Finally, further work can also be conducted to optimize purification yields, for example, by adjusting the growing media.

Using human sera from CDI-recovered patients, we confirmed that these recombinant toxins could be used as antigens for ELISA for our studies based on a high correlation between the quantitative results of anti-TcdA and anti-TcdB antibodies obtained with recombinant and native toxins. In addition, our purified recombinant toxins conserved their biological activities compared to native toxins. Indeed, using a cell cytotoxicity assay on Vero cells, we observed a dose-dependent cytopathic activity for both recombinant toxins, and this activity can be neutralized using antibodies from a CDI-recovered patient. Moreover, both recombinant toxins alter the structure of intestinal epithelia by modifying the actin cytoskeleton and opening tight junctions, as already described [15,39,40]. While we obtained similar activities between our recombinant toxins and the native VPI 10463 toxins, the concentrations used in our work are higher than those used in previous publications [41]. Importantly, the cytotoxic effect of native and recombinant toxins was observed at lower concentrations, down to 0.8 µg.mL^−1^. This could be due to various parameters, including the long-term storage of the native VPI 10463 toxins.

Compared to other available methods, the method we present has the following limitations and advantages: The first limitation is that using *C. difficile* as the native host implies that researchers have to have access to both a BSL2 laboratory and equipment enabling anaerobic work. Second, the yield obtained, while reasonable for proteins of such sizes, remains limited. This could be due to the fact that the toxins are not secreted in our experimental conditions. However, recent studies have shown that toxin secretion varies across *C. difficile* strains [37]. Indeed, the 630 strain used in our work appears to be the strain for which toxin secretion was the lowest [37]. Furthermore, toxins are secreted during the transition between exponential and stationary phases, while our work uses a short induction step during exponential growth. Finally, secretion could also be hindered by the presence of a His-tag, preventing secretion. Authors interested in getting these toxins secreted could either use a strain background associated with high physiological secretion of toxins, such as the UK1 or VPI 10463 strains [37], or genetically modify the 630 strain to increase toxin secretion.

However, there are several advantages to the method presented in our work. First, our method is quick and uses routinely available materials. Indeed, after optimizing the production and purification processes, we were able to shorten toxin expression time by 10 (a half day instead of five days) and simplify the purification process compared to native toxins production. Second, this method relies on using the native host, which minimizes the risk of having variations in post-translational modifications of the recombinant proteins as compared to recombinant proteins obtained in heterologous hosts. Third, our method relies on modification of the genes directly in their native positions in the bacterial chromosome. This gives the opportunity to keep the native genetic environment of the genes of interest. In addition, it removes the need for antibiotic maintenance of plasmids while ensuring stability over time. Furthermore, plasmids used to genetically engineer the chromosome rely on smaller DNA inserts as compared to plasmids carrying whole toxin genes, which simplifies the cloning steps. Finally, our work provides proof of the concept that the toxin locus can be genetically engineered in situ to produce recombinant tagged toxins while maintaining biological activity. Indeed, using the 630Δ*erm* as a host strain allows for flexibility in genetic engineering as compared to strains such as the VPI 10463. While we decided to use His-tags for the purification of those toxins, researchers could be interested in using other types of protein tags, such as the SNAP tag, which allows visualization of proteins by microscopy. This strategy could also be extended to the production of other modified toxins. For instance, authors interested in studying the contribution of various toxins and subtypes during infection could engineer *C. difficile* strains expressing truncated toxins and study those strains in in vivo infection models. This is particularly crucial as new data emerges on the subtypes of TcdA and TcdB toxins and their respective roles. Indeed, it now appears that we can no longer consider TcdA and TcdB as two individual toxins homogenous across strains but rather as groups of toxin subtypes with variations in structures and biological targets. For instance, TcdB2 and TcdB4 toxin subtypes have been shown to be recognized by the TFPI receptor, while TcdB1/TcdB3/TcdB5 appear to recognize Wnt receptor Frizzled proteins (FZD). Since toxins from the 630Δ*erm* or VPI 10463 strains appear in distinct clusters from toxins of the R20291 strain (Subtypes A1/A2 and B1/B2, respectively), toxins purified from the 630Δ*erm*/VPI 10463 strains would not be adequate proxies for the study of toxins from the R20291 strain or any strain possessing TcdB2 toxins. As such, researchers interested in deciphering the roles of TcdA and TcdB will require tools that allow for the construction, production, and characterization of specific toxin subtypes in their native hosts. To the best of our knowledge, such flexibility in toxin sources and production was unattainable until now.

Through this work, we successfully generated strains able to produce recombinant toxins A and B of *C. difficile* on demand and showed that the recombinant toxins obtained have similar biological activities compared to native toxins. Simple genetic engineering is therefore all that is needed for the production of toxins in the 630Δ*erm* strain background, from the production of TcdA and/or TcdB from any *C. difficile* strain to the construction of truncated or chimeric toxins. We believe that these purified toxins and genetically engineered strains will be valuable and helpful tools used to better understand the pathogenesis of *C. difficile* infections.

## 4. Materials and Methods

### 4.1. Bacterial Strains, Plasmids, and Growth Conditions

Bacterial strains, primers, and plasmids used in this study are detailed in Tables S1–S3. *E. coli* strains were grown aerobically at 37 °C in Luria Bertani (LB) medium (Becton, Dickinson, MD, USA) supplemented with Ampicillin (Amp; 100 µg.mL^−1^) or Chloramphenicol (Cm; 25 µg.mL^−1^) as appropriate. *C. difficile* strains were grown at 37 °C in an anaerobic chamber using Brain Heart Infusion (BHI) medium (Becton, Dickinson, MD, USA) or tryptone-yeast medium (TY; 3% Bacto tryptose (Becton, Dickinson, MD, USA), 2% Bacto yeast extract (Becton, Dickinson, MD, USA), and 0.1% thioglycolate, adjusted to pH 7.4) supplemented as appropriate with Thiamphenicol (Tm; 15 μg.mL^−1^), Aztreonam (Az; 16 μg.mL^−1^) or nonantibiotic analog Anhydro—tetracycline (ATc; 0.05 µg.mL^−1^, 0.1 µg.mL^−1^, 0.25 µg.mL^−1^, 0.5 µg.mL^−1^).

### 4.2. Constructs and Cloning in the C. difficile 630∆erm Strain

Plasmid extraction (Omega, VWR, Radnor, PA, USA), endonuclease digestion (New England Biolabs, Ipswich, MA, USA), ligation (New England Biolabs, Ipswich, MA, USA), and agarose gel electrophoresis were carried out as described by Maniatis et al. [42]. The Phusion polymerase (New England Biolabs, Ipswich, MA, USA) used for Polymerase Chain Reaction (PCR) and the Golden Gate cloning kit (New England Biolabs, Ipswich, MA, USA) were carried out according to the manufacturer’s instructions. All inserts cloned in the constructed plasmids were sequenced (Eurofins Genomics, Ebersberg, Germany).

In order to have a plasmid suitable for the Golden Gate cloning strategy, pTC130 was constructed. First, the spectinomycin cassette from the pAT28 plasmid [43] was amplified using primers 2836/2837 and cloned into the pBlunt2 plasmid (Invitrogen, San Diego, CA, USA), giving rise to pTC129. The 1.09 kb BamHI/EcoRV fragment extracted from pTC129 was ligated into pMSR [44] and digested with BamHI/PvuII, giving rise to pTC130.

Plasmids pADS1 and pADS2 were constructed for the genomic insertion of 6xHis-tags before the STOP codons of *tcdA* and *tcdB*, respectively, using the following strategy: Two DNA fragments flanking the STOP codon of *tcdA* were obtained by PCR using *C. difficile* 630∆*erm* genomic DNA and primers 2854/2851 and 2852/2853. These DNA fragments were cloned into pTC130 by the golden gate method [45], giving rise to pADS1. Similarly, two PCR DNA fragments flanking the STOP codon of *tcdB* were obtained using 2858/2859 and 2860/2861 primer pairs on *C. difficile* 630∆*erm* genomic DNA and cloned into pTC130 by the golden gate method, giving rise to pADS2. Plasmids pADS1 and pADS2 were confirmed by enzymatic restriction as well as sequencing of the inserts. pADS1 and pADS2 recombinant pseudo-suicide plasmids were transferred by heterogramic conjugation from conjugative *E. coli* strain HB101 (pRK24) to *C. difficile* 630∆*erm*. The recombinant strains AD1 and AD2 were selected as described by Peltier et al. [44]. Recombinant mutants were confirmed by PCR and sequencing of the targeted genomic region.

The same strategy was used to build the pADS3 and pADS4 plasmids to replace the *tcdA* and *tcdB* promoters with a P_tet_ promoter. Homologous fragments upstream (CO1) and downstream (CO2) of the *tcdA* promoter were amplified from *C. difficile* 630∆*erm* extracted genomic DNA using primers 3046/3047 and 3050/3051. The P_tet_ promoter from the pRPF185 plasmid [46] was amplified using primers 3048/3049. These three fragments were cloned into pJV7 [47] by the golden gate method, giving rise to pADS3. Similarly, PCR DNA fragments CO1 and CO2 flanking the promoter of *tcdB,* obtained using primers 3052/3053 and 3056/3057 on *C. difficile* 630∆*erm* genomic DNA, as well as the P_tet_ promoter, amplified from the pRPF185 plasmid using primers 3054/3055, were cloned into pJV7 by the golden gate method, giving rise to pADS4. After confirmation by enzymatic restriction and sequencing, the pADS3 and pADS4 recombinant pseudo-suicide plasmids were transferred by heterogramic conjugation from conjugative *E. coli* strain HB101 (pRK24) to *C. difficile* strains AD1 (*tcdA*-His) and AD2 (*tcdB*-His). Selection of the recombinant strains AD3 (P_tet_*-tcdA*-His) and AD4 (P_tet_*-tcdB*-His) was performed as described by Peltier et al. [44]. Recombinant strains were subjected to PCR screening and sequencing to confirm the correct insertion of the fragments and the absence of additional mutations. Strains and plasmids detailed in this study are available for researchers upon request.

### 4.3. Expression and Purification of Recombinant Toxins

#### 4.3.1. From AD1 and AD2 Strains

Cultures of *C. difficile* strains AD1 and AD2 were prepared in 600 mL of TY medium, inoculated at a 600 nm optical density (OD_600nm_) of 0.05 using overnight cultures, and allowed to grow for 75 to 92 h in an anaerobic chamber at 37 °C to induce toxin release in nutrient-limiting medium [12]. Bacteria were centrifuged, and culture supernatants were precipitated with 45 g of ammonium sulfate for 100 mL of bacterial culture, followed by shaking at room temperature for 5 min. Precipitates were centrifuged (12,200× *g*) for 10 min at 4 °C, and the pellet was resuspended in 40 mL of denaturing buffer (8 M Urea, 0.1 M NaH2PO4, 0.01 M Tris-HCl, pH 8). pH was adjusted to 8 after resuspension to allow the binding of 6× His-tagged toxins to Ni-NTA agarose resin (reference: 30210; Qiagen, Hilden, Germany). The resulting samples were passed through chromatography columns containing the Ni-NTA agarose resin and eluted with denaturing buffer containing increasing concentrations of imidazole (10 mM, 20 mM, 30 mM, 40 mM, 60 mM, 80 mM, 100 mM, 200 mM, and 1 M). For analysis, fractions were loaded into a sodium dodecyl-sulfate polyacrylamide gel electrophoresis (SDS-PAGE) gel using 6% stacking gel and 10% separation gel, followed by a standard Coomassie stain.

#### 4.3.2. From AD3 and AD4 Strains

Cultures of *C. difficile* strains AD3 and AD4 were prepared in 1000 mL and 2000 mL of BHI medium, respectively, inoculated at OD_600nm_ of 0.05 using overnight cultures, and incubated in an anaerobic chamber at 37 °C. Cultures were allowed to grow until reaching an OD_600nm_ of 0.5 before toxin expression was induced by the addition of ATc (0.5 µg.mL^−1^ for recombinant toxin A rTcdA or 0.1 µg.mL^−1^ for recombinant toxin B rTcdB). Cultures were incubated for 4 h after ATc addition, bacteria were centrifuged, and pellets were frozen at −20 °C. Cell pellets were resuspended in 40 mL of lysis buffer (50 mM NaH_2_PO_4_, 300 mM NaCl, 10 mM Imidazole, pH 8). For lysis, bacteria were then incubated for one hour at 37 °C [46], followed by a brief sonication to reduce viscosity. Lysates were centrifuged (12,200× *g*) for 30 min at 4 °C. Lysate supernatants were then passed through chromatography columns containing Ni-NTA agarose resin (reference: 30210; Qiagen, Hilden, Germany). The bound 6× His-tagged toxins were eluted with elution buffer (50 mM NaH_2_PO_4_, 300 mM NaCl, 200 mM Imidazole, pH 8). The eluents were desalted using a PD10 column (reference 17085101; Cytiva, Shrewsbury, MA, USA), and the recombinant toxins were eluted with 50 mM NaH_2_PO_4_ and 300 mM NaCl and conserved at −20 °C. Samples were analyzed by SDS-PAGE as previously described. Purified proteins were obtained at 80 µg.mL^−1^ and 60 µg.mL^−1^ for rTcdA and rTcdB, respectively, on average. Quantification of protein concentrations was performed using the Bradford assay.

### 4.4. Cytotoxicity Assay

The cytotoxic activity of purified recombinant toxins rTcdA and rTcdB was determined using Vero (African green monkey kidney) cell monolayer culture with a protocol adapted from Cartman et al. [48]. Briefly, cells were seeded in a 96-well plate (TPP) with 100 µL cell suspension at a density of 2 × 10^5^ cells mL^−1^ in Dulbecco’s modified Eagle’s medium (DMEM) (GIBCO, ThermoFisher Scientific, Waltham, MA, USA) supplemented with 10% (*v*/*v*) fetal calf serum (Dutscher, Bernolsheim, France). The plates were incubated for 24 h at 37 °C with 5% CO_2_ to obtain the cell monolayer before adding the toxins. In order to evaluate the cytotoxicity of the recombinant toxins compared to native TcdA and TcdB, cells were treated with either TcdA, TcdB, rTcdA, or rTcdB using four different concentrations ranging from 4 µg.mL^−1^ to 32 ng.mL^−1^. These dilutions were obtained by preparing fivefold serial dilutions of toxin stocks in DMEM medium supplemented with 0.1% of Bovine serum albumin solution (BSA) (Sigma Aldrich, St. Louis, MO, USA). In addition, cells in DMEM and 0.1% of BSA were included as a negative control. The *C. difficile* native toxins TcdA and TcdB used in this study were produced in the *C. difficile* VPI 10463 strain and generously gifted by Pr. Michel Popoff (Pasteur Institute, Paris, 75724 France) [20,22]. After 18 h of incubation, morphological alterations of Vero cells were observed under phase contrast microscopy (Zeiss, Oberkochen, Germany). The results presented here are representative of at least two independent experiments.

### 4.5. Disruption of the Actin Cytoskeleton by Purified Recombinant Toxins

A monolayer of Vero cells was obtained by seeding 1 mL of a 1 × 10^5^ cells mL^−1^ suspension per well in a 24-well plate (TPP), followed by incubation for 24 h at 37 °C with 5% CO_2_. Sub-confluent cells were then incubated for 18 h with 4 µg.mL^−1^ of native toxins (positive controls) or purified recombinant toxins. In addition, a negative control was also added by keeping cells in DMEM and 0.1% BSA only. After incubation, cells were fixed and stained with Rhodamine Phalloidin (reference: P1951, Sigma Aldrich, St. Louis, MO, USA) and DAPI contained in the Fluoromount-G^TM^ mounting medium (reference: 00495952, Invitrogen, San Diego, CA, USA). Five random fields were acquired for each condition on a LEICA SP8 confocal microscope using ×20 objective, and FIJI software version 2.9.0/1.53t was used to determine the mean area per cell [49]. For each field randomly selected, using a thresholding method and particle analysis, we used the actin channel to determine the area of the field occupied by cells, expressed in µm^2^, and the DAPI channel to count the number of nuclei in the field. We then expressed the average cell area, expressed in µm^2^/cell, by dividing the total cell area by the number of cells. Finally, additional images were acquired with the ×63 objective in order to illustrate cell morphological alterations induced by toxins.

### 4.6. Comparison of Native or Recombinant Toxins for the Quantification of Serum Antibodies by Quantitative ELISA

The TcdA and TcdB antibody titers were determined by quantitative enzyme-linked immunosorbent assay (ELISA) using a protocol adapted from Péchiné et al. and Launay et al. [50,51] and following the Clinical Microbiology Guidelines from SFM [52]. In order to allow quantitative measurements of serum antibody titers, we added a calibration range by serially diluting a polyclonal IgG antibody from human sera solution (reference: I2511, Sigma, Livonia, MI, USA) to reach concentrations ranging from 0.68 ng.mL^−1^ to 22 ng.mL^−1^. Briefly, ELISA microplates (MaxiSorp Nunc, Rochester, NY, USA, reference: 439454) were coated with 1 µg.mL^−1^ of native or purified recombinant toxins in a coating buffer (PBS—Deoxycholate 0.1%) and incubated overnight at 35 °C [52]. Microplates were washed four times by adding 250 µL of PBS-Tween 0.1% in each well. After this step, wells were incubated with 200 µL of a blocking buffer (PBS—Bovine Serum Albumin 3% (BSA reference: A9418, Sigma Aldrich, St. Louis, MO, USA) for one hour at 35 °C. After washing the blocking buffer, serum samples from *C. difficile*-recovered patients were tested in technical duplicates at serial dilutions from 1/20 to 1/2560 in dilution buffer (PBS—Tween 0.1%—BSA 0.1%). After a washing step, plates were incubated with a secondary antibody (anti-Human IgG-Phosphatase alkaline antibody (reference: A9544, Sigma Aldrich, St. Louis, MO, USA), incubated for one hour at 35 °C, and washed four times with PBS-Tween 0.1%. Finally, revelation was carried out by adding the p-nitrophenyl phosphate substrate (reference: N2770, Sigma Aldrich, St. Louis, MO, USA) to each well. The light-absorption was measured at 450 nm, and anti-TcdA or anti-TcdB IgG concentrations were determined using calibration ranges. Serum samples used in these assays were obtained from seven CDI-recovered patients from the SERODIFF study (ClinicalTrials.gov Identifier: NCT01946750, https://clinicaltrials.gov/study/NCT01946750?term=NCT01946750&rank=1, accessed on 4 September 2023). For the SERODIFF study, participants were recruited from 1 December 2012 to 30 June 2017. Written informed consent forms were collected for each participant.

### 4.7. Neutralization Antibody Assay

For the neutralization antibody assay, 96-well plates (TPP) were prepared to obtain monolayers of Vero cells using the procedure described in the cytotoxicity assay section. Antibodies used in these assays were obtained from serum samples collected from CDI-recovered patients included in the SERODIFF study. In order to confirm that the recombinant toxins were also susceptible to the neutralizing activity of these antibodies, 75 µL of a 4 µg.mL^−1^ solution of toxins was mixed with 75 µL of 1/10 to 1/160 dilutions of serum from CDI-recovered patients and incubated at 37 °C for 60 min [27,28]. The mixtures were then added to monolayers of Vero cells, and the plates were incubated in 5% CO_2_ at 37 °C for 18 h. A negative control (cells in DMEM and 0.1% of BSA only) and positive controls (4 µg.mL^−1^ and 0.8 µg.mL^−1^ of rTcdA or rTcdB alone) were also added. Cell morphological alterations were observed under phase contrast microscopy (Zeiss, Oberkochen, Germany).

### 4.8. Phylogenetic Analysis

Phylogenetic analyses were conducted on the following 39 genomes of *C. difficile* strains: 630Δ*erm*, A685, CD015, CD196, CIP1079324, E1, E7, E12, E14, VPI 10463, E15, E16, E23, E24, E25, E28, NAP07, NAP08, QCD_23m63, QCD_32g58, QCD_37x79, QCD-63q42, QCD_66c26, QCD_76w55, QCD_97b34, T3, T5, T6, T10, T11, T15, T17, T19, T20, T22, T23, T42, T61, and R20291. Selected genomes and protein sequences were obtained from Marc Monot [53]. In addition, protein sequences of large clostridial toxins from *Paeniclostridium_sordellii* were obtained from NCBI for TcsH (AGK40891.1) and TcsL (Q46342.1). See Supplementary Table S4 for genome accession numbers.

For phylogenetic trees, protein sequences were first aligned with MUSCLE provided by EMBL-EBI (version 3.8.425, http://www.ebi.ac.uk/Tools/msa/muscle/, accessed on 25 January 2023) [54]. Phylogeny reconstruction was performed with FastTree (version 2.1.11) using a local installation and running with default settings [55]. Generated trees were exported and uploaded to iTOL (version 6, https://itol.embl.de/, accessed on 25 January 2023) for visualization [56], and bootstrap metadata were visualized using colored tree branches (red = 0/blue = 1). Sequence alignments obtained in MUSCLE were visualized using BioEdit (version 7.0.5.3) [57] to generate the amino acid alignment comparisons for TcdA and TcdB in *C. difficile* strains 630Δ*erm*, R20291, and VPI 10463.

### 4.9. Statistical Analysis and Software

Analyses were performed using GraphPad Prism 9 (GraphPad Software version 9.0.0, San Diego, CA, USA). The linear correlation was determined by the Pearson correlation coefficient. For microscopy, figure assembly was conducted using the FigureJ plugin (version 1.39) [58].

## Data Availability

The original contributions presented in the study are included in the article/supplementary material, further inquiries can be directed to the corresponding author/s.

## Supplementary Materials

The following supporting information can be downloaded at: https://www.mdpi.com/article/10.3390/toxins16010038/s1. File S1: Protein comparisons for TcdA and TcdB from the 630Δ*erm*, VPI10463, and R20291 *C. difficile* strains; File S2: Supplementary material and methods; Figure S1: Strain construction for the production of the recombinant toxins rTcdA and rTcdB of *C. difficile*; Figure S2: Purification of recombinant His-tagged toxins by Ni-NTA affinity chromatography under denaturing conditions from the genetic construction without the P_tet_ promoter; Figure S3: Validation of the recombinant protein extraction method; Figure S4: Disruption of the cytoskeleton in Vero cells with a 20× objective; Figure S5: Serum without anti-TcdA neutralizing IgG does not neutralize TcdA toxins activity; Figure S6: Serum with anti-TcdB-neutralizing IgG neutralizes both native and recombinant TcdB activity; Table S1: List of bacterial strains used in this study; Table S2: List of Primers used in this study; Table S3: List of Plasmids used in this study; Table S4: Table List of Genome Accession Numbers Used for Figure 1. References [43,46,47,59] are cited in the supplementary materials.
