# Supplementary material for: A Streamlined Method to Obtain Biologically Active TcdA and TcdB Toxins from Clostridioides difficile
Source: Toxins (Basel). 2024 Jan 11;16(1):38. doi: 10.3390/toxins16010038 (PMC10821508; doi:10.3390/toxins16010038)
Supplement: Supplementary file 1 [file toxins-16-00038-s001.zip › File S1.pdf]

Sequence Identity Matrix

TcdA

	tcdA_630erm	tcdA_VPI10463	tcdA_R20291
tcdA_630erm	ID	0,998	0,981
tcdA_VPI10463	0,998	ID	0,981
tcdA_R20291	0,981	0,981	ID

TcdB

	tcdB_630erm	tcdB_VPI10463	tcdB_R20291
tcdB_630erm	ID	1,000	0,921
tcdB_VPI10463	1,000	ID	0,921
tcdB_R20291	0,921	0,921	ID

```
                          10         20         30         40         50
                 ....|....|....|....|....|....|....|....|....|....|
tcdA_630erm      MSLISKEELIKLAYSIRPRENEYKTILTNLDEYNKLTTNNNENKYLQLKK
tcdA_VPI10463    ..................................................
tcdA_R20291      ..................................................

                          60         70         80         90        100
                 ....|....|....|....|....|....|....|....|....|....|
tcdA_630erm      LNESIDVFMNKYKTSSRNRALSNLKKDILKEVILIKNSNTSPVEKNLHFV
tcdA_VPI10463    ..................................................
tcdA_R20291      .............N....................................

                         110        120        130        140        150
                 ....|....|....|....|....|....|....|....|....|....|
tcdA_630erm      WIGGEVSDIALEYIKQWADINAEYNIKLWYDSEAFLVNTLKKAIVESSTT
tcdA_VPI10463    ..................................................
tcdA_R20291      ..................................................

                         160        170        180        190        200
                 ....|....|....|....|....|....|....|....|....|....|
tcdA_630erm      EALQLLEEEIQNPQFDNMKFYKKRMEFIYDRQKRFINYYKSQINKPTVPT
tcdA_VPI10463    ..................................................
tcdA_R20291      ..................................................

                         210        220        230        240        250
                 ....|....|....|....|....|....|....|....|....|....|
tcdA_630erm      IDDIIKSHLVSEYNRDETVLESYRTNSLRKINSNHGIDIRANSLFTEQEL
tcdA_VPI10463    ..................................................
tcdA_R20291      ..................L...............................

                         260        270        280        290        300
                 ....|....|....|....|....|....|....|....|....|....|
tcdA_630erm      LNIYSQELLNRGNLAAASDIVRLLALKNFGGVYLDVDMLPGIHSDLFKTI
tcdA_VPI10463    ..................................................
tcdA_R20291      ..................................................

                         310        320        330        340        350
                 ....|....|....|....|....|....|....|....|....|....|
tcdA_630erm      SRPSSIGLDRWEMIKLEAIMKYKKYINNYTSENFDKLDQQLKDNFKLIIE
tcdA_VPI10463    ..................................................
tcdA_R20291      P.................................................

                         360        370        380        390        400
                 ....|....|....|....|....|....|....|....|....|....|
tcdA_630erm      SKSEKSEIFSKLENLNVSDLEIKIAFALGSVINQALISKQGSYLTNLVIE
tcdA_VPI10463    ..................................................
tcdA_R20291      ..................................................

                         410        420        430        440        450
                 ....|....|....|....|....|....|....|....|....|....|
tcdA_630erm      QVKNRYQFLNQHLNPAIESDNNFTDTTKIFHDSLFNSATAENSMFLTKIA
tcdA_VPI10463    ..................................................
tcdA_R20291      ..................................................

                         460        470        480        490        500
                 ....|....|....|....|....|....|....|....|....|....|
tcdA_630erm      PYLQVGFMPEARSTISLSGPGAYASAYYDFINLQENTIEKTLKASDLIEF
tcdA_VPI10463    ..................................................
tcdA_R20291      ..................................................

                         510        520        530        540        550
                 ....|....|....|....|....|....|....|....|....|....|
tcdA_630erm      KFPENNLSQLTEQEINSLWSFDQASAKYQFEKYVRDYTGGSLSEDNGVDF
tcdA_VPI10463    ..................................................
tcdA_R20291      ..................................................
```

```
                                   560                 570                 580                 590                 600
                   . . . . | . . . . | . . . . | . . . . | . . . . | . . . . | . . . . | . . . . | . . . . | . . . . |
tcdA_630erm        N K N T A L D K N Y L L N N K I P S N N V E E A G S K N Y V H Y I I Q L Q G D D I S Y E A T C N L F
tcdA_VPI10463      . . . . . . . . . . . . . . . . . . . . . . . . . . . . . . . . . . . . . . . . . . . . . . . . . .
tcdA_R20291        . . . . . . . . . . . . . . . . . . . . . . . . . . . . . . . . . . . . . . . . . . . . . . . . . .

                                   610                 620                 630                 640                 650
                   . . . . | . . . . | . . . . | . . . . | . . . . | . . . . | . . . . | . . . . | . . . . | . . . . |
tcdA_630erm        S K N P K N S I I I Q R N M N E S A K S Y F L S D D G E S I L E L N K Y R I P E R L K N K E K V K V
tcdA_VPI10463      . . . . . . . . . . . . . . . . . . . . . . . . . . . . . . . . . . . . . . . . . . . . . . . . . .
tcdA_R20291        . . . . . . . . . . . . . . . . . . . . . . . . . . . . . . . . . . . . . . . . . . . . . . . . . .

                                   660                 670                 680                 690                 700
                   . . . . | . . . . | . . . . | . . . . | . . . . | . . . . | . . . . | . . . . | . . . . | . . . . |
tcdA_630erm        T F I G H G K D E F N T S E F A R L S V D S L S N E I S S F L D T I K L D I S P K N V E V N L L G C
tcdA_VPI10463      . . . . . . . . . . . . . . . . . . . . . . . . . . . . . . . . . . . . . . . . . . . . . . . . . .
tcdA_R20291        . . . . . . . . . . . . . . . . . . . . . . . . . . . . . . . . . . . . . . . . . . . . . . . . . .

                                   710                 720                 730                 740                 750
                   . . . . | . . . . | . . . . | . . . . | . . . . | . . . . | . . . . | . . . . | . . . . | . . . . |
tcdA_630erm        N M F S Y D F N V E E T Y P G K L L L S I M D K I T S T L P D V N K N S I T I G A N Q Y E V R I N S
tcdA_VPI10463      . . . . . . . . . . . . . . . . . . . . . . . . . . . . . . . . . . . . . . . . . . . . . . . . . .
tcdA_R20291        . . . . . . . . . . . . . . . . . . . . . . . . . . . . . . . . . . D . . . . . . . . . . . . . . .

                                   760                 770                 780                 790                 800
                   . . . . | . . . . | . . . . | . . . . | . . . . | . . . . | . . . . | . . . . | . . . . | . . . . |
tcdA_630erm        E G R K E L L A H S G K W I N K E E A I M S D L S S K E Y I F F D S I D N K L K A K S K N I P G L A
tcdA_VPI10463      . . . . . . . . . . . . . . . . . . . . . . . . . . . . . . . . . . . . . . . . . . . . . . . . . .
tcdA_R20291        . . . . . . . . . . . . . . . . . . . . . . . . . . . . . . . . . . . . . . . . . . . . . . . . . .

                                   810                 820                 830                 840                 850
                   . . . . | . . . . | . . . . | . . . . | . . . . | . . . . | . . . . | . . . . | . . . . | . . . . |
tcdA_630erm        S I S E D I K T L L L D A S V S P D T K F I L N N L K L N I E S S I G D Y I Y Y E K L E P V K N I I
tcdA_VPI10463      . . . . . . . . . . . . . . . . . . . . . . . . . . . . . . . . . . . . . . . . . . . . . . . . . .
tcdA_R20291        . . . . . . . . . . . . . . . . . . . . . . . . . . . . . . . . . . . . . . . . . . . . . . . . . .

                                   860                 870                 880                 890                 900
                   . . . . | . . . . | . . . . | . . . . | . . . . | . . . . | . . . . | . . . . | . . . . | . . . . |
tcdA_630erm        H N S I D D L I D E F N L L E N V S D E L Y E L K K L N N L D E K Y L I S F E D I S K N N S T Y S V
tcdA_VPI10463      . . . . . . . . . . . . . . . . . . . . . . . . . . . . . . . . . . . . . . . . . . . . . . . . . .
tcdA_R20291        . . . . . . . . . . . . . . . . . . . . . . . . . . . . . . . . . . . . . . . . . . . . . . . . . .

                                   910                 920                 930                 940                 950
                   . . . . | . . . . | . . . . | . . . . | . . . . | . . . . | . . . . | . . . . | . . . . | . . . . |
tcdA_630erm        R F I N K S N G E S V Y V E T E K E I F S K Y S E H I T K E I S T I K N S I I T D V N G N L L D N I
tcdA_VPI10463      . . . . . . . . . . . . . . . . . . . . . . . . . . . . . . . . . . . . . . . . . . . . . . . . . .
tcdA_R20291        . . . . . . . . . . . . . . . . . . . . . . . . . . . . . . . . . . . . . . . . . . . . . . . . . .

                                   960                 970                 980                 990                1000
                   . . . . | . . . . | . . . . | . . . . | . . . . | . . . . | . . . . | . . . . | . . . . | . . . . |
tcdA_630erm        Q L D H T S Q V N T L N A A F F I Q S L I D Y S S N K D V L N D L S T S V K V Q L Y A Q L F S T G L
tcdA_VPI10463      . . . . . . . . . . . . . . . . . . . . . . . . . . . . . . . . . . . . . . . . . . . . . . . . . .
tcdA_R20291        . . . . . . . . . . . . . . . . . . . . . . . . . . . . . . . . . . . . . . . . . . . . . . . . . .

                                  1010                1020                1030                1040                1050
                   . . . . | . . . . | . . . . | . . . . | . . . . | . . . . | . . . . | . . . . | . . . . | . . . . |
tcdA_630erm        N T I Y D S I Q L V N L I S N A V N D T I N V L P T I T E G I P I V S T I L D G I N L G A A I K E L
tcdA_VPI10463      . . . . . . . . . . . . . . . . . . . . . . . . . . . . . . . . . . . . . . . . . . . . . . . . . .
tcdA_R20291        . . . . . . . . . . . . . . . . . . . . . . . . . . . . . . . . . . . . . . . . . . . . . . . . . .

                                  1060                1070                1080                1090                1100
                   . . . . | . . . . | . . . . | . . . . | . . . . | . . . . | . . . . | . . . . | . . . . | . . . . |
tcdA_630erm        L D E H D P L L K K E L E A K V G V L A I N M S L S I A A T V A S I V G I G A E V T I F L L P I A G
tcdA_VPI10463      . . . . . . . . . . . . . . . . . . . . . . . . . . . . . . . . . . . . . . . . . . . . . . . . . .
```

```
tcdA_R20291   ..................................................

                  1110      1120      1130      1140      1150
              ....|....|....|....|....|....|....|....|....|....|
tcdA_630erm   ISAGIPSLVNNELILHDKATSVVNYFNHLSESKKYGPLKTEDDKILVPID
tcdA_VPI10463 ..................................................
tcdA_R20291   .................................E................

                  1160      1170      1180      1190      1200
              ....|....|....|....|....|....|....|....|....|....|
tcdA_630erm   DLVISEIDFNNNSIKLGTCNILAMEGGSGHTVTGNIDHFFSSPSISSHIP
tcdA_VPI10463 ..................................................
tcdA_R20291   ...........................................Y......

                  1210      1220      1230      1240      1250
              ....|....|....|....|....|....|....|....|....|....|
tcdA_630erm   SLSIYSAIGIETENLDFSKKIMMLPNAPSRVFWWETGAVPGLRSLENDGT
tcdA_VPI10463 ..................................................
tcdA_R20291   ...V......K....................................N..

                  1260      1270      1280      1290      1300
              ....|....|....|....|....|....|....|....|....|....|
tcdA_630erm   RLLDSIRDLYPGKFYWRFYAFFDYAITTLKPVYEDTNIKIKLDKDTRNFI
tcdA_VPI10463 ..................................................
tcdA_R20291   K....................................T............

                  1310      1320      1330      1340      1350
              ....|....|....|....|....|....|....|....|....|....|
tcdA_630erm   MPTITTNEIRNKLSYSFDGAGGTYSLLLSSYPISTNINLSKDDLWIFNID
tcdA_VPI10463 ..................................................
tcdA_R20291   ......D...........................M...............

                  1360      1370      1380      1390      1400
              ....|....|....|....|....|....|....|....|....|....|
tcdA_630erm   NEVREISIENGTIKKGKLIKDVLSKIDINKNKLIIGNQTIDFSGDIDNKD
tcdA_VPI10463 ..................................................
tcdA_R20291   ................N..E..............................

                  1410      1420      1430      1440      1450
              ....|....|....|....|....|....|....|....|....|....|
tcdA_630erm   RYIFLTCELDDKISLIIEINLVAKSYSLLLSGDKNYLISNLSNIIEKINT
tcdA_VPI10463 ...........................................T......
tcdA_R20291   ...........................................T......

                  1460      1470      1480      1490      1500
              ....|....|....|....|....|....|....|....|....|....|
tcdA_630erm   LGLDSKNIAYNYTDESNNKYFGAISKTSQKSIIHYKKDSKNILEFYNDST
tcdA_VPI10463 ..................................................
tcdA_R20291   ...............................................G..

                  1510      1520      1530      1540      1550
              ....|....|....|....|....|....|....|....|....|....|
tcdA_630erm   LEFNSKDFIAEDINVFMKDDINTITGKYYVDNNTDKSIDFSISLVSKNQV
tcdA_VPI10463 ..................................................
tcdA_R20291   ..................................................

                  1560      1570      1580      1590      1600
              ....|....|....|....|....|....|....|....|....|....|
tcdA_630erm   KVNGLYLNESVYSSYLDFVKNSDGHHNTSNFMNLFLDNISFWKLFGFENI
tcdA_VPI10463 ..................................................
tcdA_R20291   ....................................N.............

                  1610      1620      1630      1640      1650
              ....|....|....|....|....|....|....|....|....|....|
tcdA_630erm   NFVIDKYFTLVGKTNLGYVEFICDNNKNIDIYFGEWKTSSSKSTIFSGNG
```

```
tcdA_VPI10463  ..................................................
tcdA_R20291    ..................................................

                        1660      1670      1680      1690      1700
               ....|....|....|....|....|....|....|....|....|....|
tcdA_630erm    RNVVVEPIYNPDTGEDISTSLDFSYEPLYGIDRYINKVLIAPDLYTSLIN
tcdA_VPI10463  ..................................................
tcdA_R20291    ..................................................

                        1710      1720      1730      1740      1750
               ....|....|....|....|....|....|....|....|....|....|
tcdA_630erm    INTNYYSNEYYPEIIVLNPNTFHKKVNINLDSSSFEYKWSTEGSDFILVR
tcdA_VPI10463  ..................................................
tcdA_R20291    ..................................................

                        1760      1770      1780      1790      1800
               ....|....|....|....|....|....|....|....|....|....|
tcdA_630erm    YLEESNKKILQKIRIKGILSNTQSFNKMSIDFKDIKKLSLGYIMSNFKSF
tcdA_VPI10463  ..................................................
tcdA_R20291    ..................................................

                        1810      1820      1830      1840      1850
               ....|....|....|....|....|....|....|....|....|....|
tcdA_630erm    NSENELDRDHLGFKIIDNKTYYYDEDSKLVKGLININNSLFYFDPIEFNL
tcdA_VPI10463  ..................................................
tcdA_R20291    ...............................................S..

                        1860      1870      1880      1890      1900
               ....|....|....|....|....|....|....|....|....|....|
tcdA_630erm    VTGWQTINGKKYYFDINTGAALISYKIINGKHFYFNNDGVMQLGVFKGPD
tcdA_VPI10463  ......................T...........................
tcdA_R20291    .....................ST..............N............

                        1910      1920      1930      1940      1950
               ....|....|....|....|....|....|....|....|....|....|
tcdA_630erm    GFEYFAPANTQNNNIEGQAIVYQSKFLTLNGKKYYFDNDSKAVTGWRIIN
tcdA_VPI10463  ......................................N...........
tcdA_R20291    ..................................................

                        1960      1970      1980      1990      2000
               ....|....|....|....|....|....|....|....|....|....|
tcdA_630erm    NEKYYFNPNNAIAAVGLQVIDNNKYYFNPDTAIISKGWQTVNGSRYYFDT
tcdA_VPI10463  ..................................................
tcdA_R20291    ..................................................

                        2010      2020      2030      2040      2050
               ....|....|....|....|....|....|....|....|....|....|
tcdA_630erm    DTAIAFNGYKTIDGKHFYFDSDCVVKIGVFSTSNGFEYFAPANTYNNNIE
tcdA_VPI10463  ..................................................
tcdA_R20291    ...............................G..................

                        2060      2070      2080      2090      2100
               ....|....|....|....|....|....|....|....|....|....|
tcdA_630erm    GQAIVYQSKFLTLNGKKYYFDNNSKAVTGWQTIDSKKYYFNTNTAEAATG
tcdA_VPI10463  ..................................................
tcdA_R20291    ..................................................

                        2110      2120      2130      2140      2150
               ....|....|....|....|....|....|....|....|....|....|
tcdA_630erm    WQTIDGKKYYFNTNTAEAATGWQTIDGKKYYFNTNTAIASTGYTIINGKH
tcdA_VPI10463  ..................................................
tcdA_R20291    ....................................S............Y

                        2160      2170      2180      2190      2200
               ....|....|....|....|....|....|....|....|....|....|
```

```
tcdA_630erm     FYFNTDGIMQIGVFKGPNGFEYFAPANTDANNIEGQAILYQNEFLTLNGK
tcdA_VPI10463   ..................................................
tcdA_R20291     ...............V............HN............K.......

                         2210      2220      2230      2240      2250
                ....|....|....|....|....|....|....|....|....|....|
tcdA_630erm     KYYFGSDSKAVTGWRIINNKKYYFNPNNAIAAIHLCTINNDKYYFSYDGI
tcdA_VPI10463   ..................................................
tcdA_R20291     ..........I...QT.DG.............T.................

                         2260      2270      2280      2290      2300
                ....|....|....|....|....|....|....|....|....|....|
tcdA_630erm     LQNGYITIERNNFYFDANNESKMVTGVFKGPNGFEYFAPANTHNNNIEGQ
tcdA_VPI10463   ..................................................
tcdA_R20291     ..................................................

                         2310      2320      2330      2340      2350
                ....|....|....|....|....|....|....|....|....|....|
tcdA_630erm     AIVYQNKFLTLNGKKYYFDNDSKAVTGWQTIDGKKYYFNLNTAEAATGWQ
tcdA_VPI10463   ..................................................
tcdA_R20291     ................................S..........V.V....

                         2360      2370      2380      2390      2400
                ....|....|....|....|....|....|....|....|....|....|
tcdA_630erm     TIDGKKYYFNLNTAEAATGWQTIDGKKYYFNTNTFIASTGYTSINGKHFY
tcdA_VPI10463   ..................................................
tcdA_R20291     ....E.....................R.......Y.......I.......

                         2410      2420      2430      2440      2450
                ....|....|....|....|....|....|....|....|....|....|
tcdA_630erm     FNTDGIMQIGVFKGPNGFEYFAPANTHNNNIEGQAILYQNKFLTLNGKKY
tcdA_VPI10463   ..........................DA......................
tcdA_R20291     ...............D..................................

                         2460      2470      2480      2490      2500
                ....|....|....|....|....|....|....|....|....|....|
tcdA_630erm     YFGSDSKAVTGLRTIDGKKYYFNTNTAVAVTGWQTINGKKYYFNTNTSIA
tcdA_VPI10463   ..................................................
tcdA_R20291     ...............................................Y..

                         2510      2520      2530      2540      2550
                ....|....|....|....|....|....|....|....|....|....|
tcdA_630erm     STGYTIISGKHFYFNTDGIMQIGVFKGPDGFEYFAPANTDANNIEGQAIR
tcdA_VPI10463   ..................................................
tcdA_R20291     ..................................................

                         2560      2570      2580      2590      2600
                ....|....|....|....|....|....|....|....|....|....|
tcdA_630erm     YQNRFLYLHDNIYYFGNNSKAATGWVTIDGNRYYFEPNTAMGANGYKTID
tcdA_VPI10463   ..................................................
tcdA_R20291     .................D.......A........................

                         2610      2620      2630      2640      2650
                ....|....|....|....|....|....|....|....|....|....|
tcdA_630erm     NKNFYFRNGLPQIGVFKGSNGFEYFAPANTDANNIEGQAIRYQNRFLHLL
tcdA_VPI10463   ..................................................
tcdA_R20291     ..................P................D..............

                         2660      2670      2680      2690      2700
                ....|....|....|....|....|....|....|....|....|....|
tcdA_630erm     GKIYYFGNNSKAVTGWQTINGKVYYFMPDTAMAAAGGLFEIDGVIYFFGV
tcdA_VPI10463   ..................................................
tcdA_R20291     ....................S.............................

                         2710
```

	.	.	.	.	|	.	.	.	.	|
tcdA_630erm	D	G	V	K	A	P	G	I	Y	G
tcdA_VPI10463	.	.	.	.	.	.	.	.	.	.
tcdA_R20291	.	.	.	.	.	.	.	.	.	.

```
                                       10                  20                  30                  40                  50
tcdB_630erm        M S L V N R K Q L E K M A N V R F R T Q E D E Y V A I L D A L E E Y H N M S E N T V V E K Y L K L K
tcdB_VPI10463      . . . . . . . . . . . . . . . . . . . . . . . . . . . . . . . . . . . . . . . . . . . . . . . . . .
tcdB_R20291        . . . . . . . . . . . . . . . . . . V . . . . . . . . . . . . . . . . . . . . . . . . . . . . . . .
Clustal Consensus  * * * * * * * * * * * * * * * * * * . * * * * * * * * * * * * * * * * * * * * * * * * * * * * * *

                                       60                  70                  80                  90                 100
tcdB_630erm        D I N S L T D I Y I D T Y K K S G R N K A L K K F K E Y L V T E V L E L K N N N L T P V E K N L H F
tcdB_VPI10463      . . . . . . . . . . . . . . . . . . . . . . . . . . . . . . . . . . . . . . . . . . . . . . . . . .
tcdB_R20291        . . . . . . . . . . . . . . . . . . . . . . . . . . . . . . . . . . . . . . . . . . . . . . . . . .
Clustal Consensus  * * * * * * * * * * * * * * * * * * * * * * * * * * * * * * * * * * * * * * * * * * * * * * * * * *

                                      110                 120                 130                 140                 150
tcdB_630erm        V W I G G Q I N D T A I N Y I N Q W K D V N S D Y N V N V F Y D S N A F L I N T L K K T V V E S A I
tcdB_VPI10463      . . . . . . . . . . . . . . . . . . . . . . . . . . . . . . . . . . . . . . . . . . . . . . . . . .
tcdB_R20291        . . . . . . . . . . . . . . . . . . . . . . . . . . . . . . . . . . . . . . . . . . . . I . . . . T
Clustal Consensus  * * * * * * * * * * * * * * * * * * * * * * * * * * * * * * * * * * * * * * * * * * * * : * * * *

                                      160                 170                 180                 190                 200
tcdB_630erm        N D T L E S F R E N L N D P R F D Y N K F F R K R M E I I Y D K Q K N F I N Y Y K A Q R E E N P E L
tcdB_VPI10463      . . . . . . . . . . . . . . . . . . . . . . . . . . . . . . . . . . . . . . . . . . . . . . . . . .
tcdB_R20291        . . . . . . . . . . . . . . . . . . . . . Y . . . . . . . . . . . . . . . . . . . T . . . . . . D .
Clustal Consensus  * * * * * * * * * * * * * * * * * * * * * : * * * * * * * * * * * * * * * * * * * : * * * * * * : *

                                      210                 220                 230                 240                 250
tcdB_630erm        I I D D I V K T Y L S N E Y S K E I D E L N T Y I E E S L N K I T Q N S G N D V R N F E E F K N G E
tcdB_VPI10463      . . . . . . . . . . . . . . . . . . . . . . . . . . . . . . . . . . . . . . . . . . . . . . . . . .
tcdB_R20291        . . . . . . . I . . . . . . . . D . . . . . S . . . . . . . . V . E . . . . . . . . . . . . . . G . .
Clustal Consensus  * * * * * * *   * * * * * * * * : * * * * * : * * * * * * * * : * : * * * * * * * * * * * * * . * *

                                      260                 270                 280                 290                 300
tcdB_630erm        S F N L Y E Q E L V E R W N L A A A S D I L R I S A L K E I G G M Y L D V D M L P G I Q P D L F E S
tcdB_VPI10463      . . . . . . . . . . . . . . . . . . . . . . . . . . . . . . . . . . . . . . . . . . . . . . . . . .
tcdB_R20291        . . K . . . . . . . . . . . . . . . . . . . . . . . . . . V . . V . . . . . . . . . . . . . . . . .
Clustal Consensus  * * : * * * * * * * * * * * * * * * * * * * * * * * * * * : * * : * * * * * * * * * * * * * * * * *

                                      310                 320                 330                 340                 350
tcdB_630erm        I E K P S S V T V D F W E M T K L E A I M K Y K E Y I P E Y T S E H F D M L D E E V Q S S F E S V L
tcdB_VPI10463      . . . . . . . . . . . . . . . . . . . . . . . . . . . . . . . . . . . . . . . . . . . . . . . . . .
tcdB_R20291        . . . . . . . . . . . . . . V . . . . . . . . . . . . . G . . . . . . . . . . . . . . . . . . . . .
Clustal Consensus  * * * * * * * * * * * * * * . * * * * * * * * * * * * *   * * * * * * * * * * * * * * * * * * * * *

                                      360                 370                 380                 390                 400
tcdB_630erm        A S K S D K S E I F S S L G D M E A S P L E V K I A F N S K G I I N Q G L I S V K D S Y C S N L I V
tcdB_VPI10463      . . . . . . . . . . . . . . . . . . . . . . . . . . . . . . . . . . . . . . . . . . . . . . . . . .
tcdB_R20291        . . . . . . . . . . . . . . . . . . . . . . . . . . . . . . . . . . . . . . . . . . . . . . . . . .
Clustal Consensus  * * * * * * * * * * * * * * * * * * * * * * * * * * * * * * * * * * * * * * * * * * * * * * * * * *

                                      410                 420                 430                 440                 450
tcdB_630erm        K Q I E N R Y K I L N N S L N P A I S E D N D F N T T T N T F I D S I M A E A N A D N G R F M M E L
tcdB_VPI10463      . . . . . . . . . . . . . . . . . . . . . . . . . . . . . . . . . . . . . . . . . . . . . . . . . .
tcdB_R20291        . . . . . . . . . . . . . . . . . . . . . . . . . . . . . A . . . . . . . . . . . . . . . . . . . .
Clustal Consensus  * * * * * * * * * * * * * * * * * * * * * * * * * * * * * : * * * * * * * * * * * * * * * * * * * *

                                      460                 470                 480                 490                 500
```

```
tcdB_630erm        GKYLRVGFFPDVKTTINLSGPEAYAAAYQDLLMFKEGSMNIHLIEADLRN
tcdB_VPI10463      ..................................................
tcdB_R20291        ..................................................
Clustal Consensus  **************************************************

                            510       520       530       540       550
                   ....|....|....|....|....|....|....|....|....|....|
tcdB_630erm        FEISKTNISQSTEQEMASLWSFDDARAKAQFEEYKRNYFEGSLGEDDNLD
tcdB_VPI10463      ..................................................
tcdB_R20291        ...................................K..............
Clustal Consensus  ***********************************.**************

                            560       570       580       590       600
                   ....|....|....|....|....|....|....|....|....|....|
tcdB_630erm        FSQNIVVDKEYLLEKISSLARSSERGYIHYIVQLQGDKISYEAACNLFAK
tcdB_VPI10463      ..................................................
tcdB_R20291        ....T.............................................
Clustal Consensus  **** *********************************************

                            610       620       630       640       650
                   ....|....|....|....|....|....|....|....|....|....|
tcdB_630erm        TPYDSVLFQKNIEDSEIAYYYNPGDGEIQEIDKYKIPSIISDRPKIKLTF
tcdB_VPI10463      ..................................................
tcdB_R20291        ..................................................
Clustal Consensus  **************************************************

                            660       670       680       690       700
                   ....|....|....|....|....|....|....|....|....|....|
tcdB_630erm        IGHGKDEFNTDIFAGFDVDSLSTEIEAAIDLAKEDISPKSIEINLLGCNM
tcdB_VPI10463      ..................................................
tcdB_R20291        ...............L..........T.......................
Clustal Consensus  ***************:**********:***********************

                            710       720       730       740       750
                   ....|....|....|....|....|....|....|....|....|....|
tcdB_630erm        FSYSINVEETYPGKLLLKVKDKISELMPSISQDSIIVSANQYEVRINSEG
tcdB_VPI10463      ..................................................
tcdB_R20291        ....V............R....V...........................
Clustal Consensus  ****:************.****:***************************

                            760       770       780       790       800
                   ....|....|....|....|....|....|....|....|....|....|
tcdB_630erm        RRELLDHSGEWINKEESIIKDISSKEYISFNPKENKITVKSKNLPELSTL
tcdB_VPI10463      ..................................................
tcdB_R20291        .....................................I............
Clustal Consensus  ************************************* ************

                            810       820       830       840       850
                   ....|....|....|....|....|....|....|....|....|....|
tcdB_630erm        LQEIRNNSNSSDIELEEKVMLTECEINVISNIDTQIVEERIEEAKNLTSD
tcdB_VPI10463      ..................................................
tcdB_R20291        .....................A..............V..G......S....
Clustal Consensus  *********************:*************:** ******.****

                            860       870       880       890       900
                   ....|....|....|....|....|....|....|....|....|....|
tcdB_630erm        SINYIKDEFKLIESISDALCDLKQQNELEDSHFISFEDISETDEGFSIRF
tcdB_VPI10463      ..................................................
tcdB_R20291        ......N............Y.........E.........L..........
Clustal Consensus  ******:************ *********:********* **********

                            910       920       930       940       950
                   ....|....|....|....|....|....|....|....|....|....|
tcdB_630erm        INKETGESIFVETEKTIFSEYANHITEEISKIKGTIFDTVNGKLVKKVNL
tcdB_VPI10463      ..................................................
```

```
tcdB_R20291          . D . . . . . . . . . . . . . A . . . . . . . . . . . . . . . . . . . . . . . . . . . . . . . . . .
Clustal Consensus    * : * * * * * * * * * * * * * : * * * * * * * * * * * * * * * * * * * * * * * * * * * * * * * * * *

                                         960                 970                 980                 990                1000
                     . . . . | . . . . | . . . . | . . . . | . . . . | . . . . | . . . . | . . . . | . . . . | . . . . |
tcdB_630erm          D T T H E V N T L N A A F F I Q S L I E Y N S S K E S L S N L S V A M K V Q V Y A Q L F S T G L N T
tcdB_VPI10463        . . . . . . . . . . . . . . . . . . . . . . . . . . . . . . . . . . . . . . . . . . . . . . . . . .
tcdB_R20291          . A . . . . . . . . . . . . . . . . . . . . . . . . . . . . . . . . . . . . . . . . . . . . . . . .
Clustal Consensus    * : * * * * * * * * * * * * * * * * * * * * * * * * * * * * * * * * * * * * * * * * * * * * * * * *

                                        1010                1020                1030                1040                1050
                     . . . . | . . . . | . . . . | . . . . | . . . . | . . . . | . . . . | . . . . | . . . . | . . . . |
tcdB_630erm          I T D A A K V V E L V S T A L D E T I D L L P T L S E G L P I I A T I I D G V S L G A A I K E L S E
tcdB_VPI10463        . . . . . . . . . . . . . . . . . . . . . . . . . . . . . . . . . . . . . . . . . . . . . . . . . .
tcdB_R20291          . . . . . . . . . . . . . . . . . . . . . . . . . . . . . . V . . . . . . . . . . . . . . . . . . .
Clustal Consensus    * * * * * * * * * * * * * * * * * * * * * * * * * * * * * * : * * * * * * * * * * * * * * * * * * *

                                        1060                1070                1080                1090                1100
                     . . . . | . . . . | . . . . | . . . . | . . . . | . . . . | . . . . | . . . . | . . . . | . . . . |
tcdB_630erm          T S D P L L R Q E I E A K I G I M A V N L T T A T T A I I T S S L G I A S G F S I L L V P L A G I S
tcdB_VPI10463        . . . . . . . . . . . . . . . . . . . . . . . . . . . . . . . . . . . . . . . . . . . . . . . . . .
tcdB_R20291          . . . . . . . . . . . . . . . . . . . . . . A . . . . . . . . . . . . . . . . . . . . . . . . . . .
Clustal Consensus    * * * * * * * * * * * * * * * * * * * * * * : * * * * * * * * * * * * * * * * * * * * * * * * * * *

                                        1110                1120                1130                1140                1150
                     . . . . | . . . . | . . . . | . . . . | . . . . | . . . . | . . . . | . . . . | . . . . | . . . . |
tcdB_630erm          A G I P S L V N N E L V L R D K A T K V V D Y F K H V S L V E T E G V F T L L D D K I M M P Q D D L
tcdB_VPI10463        . . . . . . . . . . . . . . . . . . . . . . . . . . . . . . . . . . . . . . . . . . . . . . . . . .
tcdB_R20291          . . . . . . . . . . . I . . . . . . . . . . . . S . I . . A . S . . A . . S . . . . . . . . . . . .
Clustal Consensus    * * * * * * * * * * * : * * * * * * * * * * * * . * : * * . * : * * . * *   * * * * * * * * * * * *

                                        1160                1170                1180                1190                1200
                     . . . . | . . . . | . . . . | . . . . | . . . . | . . . . | . . . . | . . . . | . . . . | . . . . |
tcdB_630erm          V I S E I D F N N N S I V L G K C E I W R M E G G S G H T V T D D I D H F F S A P S I T Y R E P H L
tcdB_VPI10463        . . . . . . . . . . . . . . . . . . . . . . . . . . . . . . . . . . . . . . . . . . . . . . . . . .
tcdB_R20291          . . . . . . . . . . . . T . . . . . . . . . . . . . . . . . . . . . . . . . . . . . . . . . . . . .
Clustal Consensus    * * * * * * * * * * * * . * * * * * * * * * * * * * * * * * * * * * * * * * * * * * * * * * * * * *

                                        1210                1220                1230                1240                1250
                     . . . . | . . . . | . . . . | . . . . | . . . . | . . . . | . . . . | . . . . | . . . . | . . . . |
tcdB_630erm          S I Y D V L E V Q K E E L D L S K D L M V L P N A P N R V F A W E T G W T P G L R S L E N D G T K L
tcdB_VPI10463        . . . . . . . . . . . . . . . . . . . . . . . . . . . . . . . . . . . . . . . . . . . . . . . . . .
tcdB_R20291          . . . . . . . . . . . . . . . . . . . . . . . . . . . . . . . . . . . . . . . . . . . . . . . . . .
Clustal Consensus    * * * * * * * * * * * * * * * * * * * * * * * * * * * * * * * * * * * * * * * * * * * * * * * * * *

                                        1260                1270                1280                1290                1300
                     . . . . | . . . . | . . . . | . . . . | . . . . | . . . . | . . . . | . . . . | . . . . | . . . . |
tcdB_630erm          L D R I R D N Y E G E F Y W R Y F A F I A D A L I T T L K P R Y E D T N I R I N L D S N T R S F I V
tcdB_VPI10463        . . . . . . . . . . . . . . . . . . . . . . . . . . . . . . . . . . . . . . . . . . . . . . . . . .
tcdB_R20291          . . . . . . . . . . . . . . . . . . . . . . . . . . . . . . . . . . . . . . . . . . . . . . . . . .
Clustal Consensus    * * * * * * * * * * * * * * * * * * * * * * * * * * * * * * * * * * * * * * * * * * * * * * * * * *

                                        1310                1320                1330                1340                1350
                     . . . . | . . . . | . . . . | . . . . | . . . . | . . . . | . . . . | . . . . | . . . . | . . . . |
tcdB_630erm          P I I T T E Y I R E K L S Y S F Y G S G G T Y A L S L S Q Y N M G I N I E L S E S D V W I I D V D N
tcdB_VPI10463        . . . . . . . . . . . . . . . . . . . . . . . . . . . . . . . . . . . . . . . . . . . . . . . . . .
tcdB_R20291          . V . . . . . . . . . . . . . . . . . . . . . . . . . . . . . . N . . . . . N . N . T . V . . . . .
Clustal Consensus    * : * * * * * * * * * * * * * * * * * * * * * * * * * * * * * * . * * * * * . * . * . * : * * * * *

                                        1360                1370                1380                1390                1400
                     . . . . | . . . . | . . . . | . . . . | . . . . | . . . . | . . . . | . . . . | . . . . | . . . . |
tcdB_630erm          V V R D V T I E S D K I K K G D L I E G I L S T L S I E E N K I I L N S H E I N F S G E V N G S N G
tcdB_VPI10463        . . . . . . . . . . . . . . . . . . . . . . . . . . . . . . . . . . . . . . . . . . . . . . . . . .
tcdB_R20291          . . . . . . . . . . . . . . . . . . . N . . . K . . . . D . . . . . D N . . . . . . . T L . . G . .
Clustal Consensus    * * * * * * * * * * * * * * * * * * * . * * * . * * * * : * * * * * : . * * * * * * *   : * * . * *
```

```
                                          1410                    1420                    1430                    1440                    1450
                   . . . . | . . . . | . . . . | . . . . | . . . . | . . . . | . . . . | . . . . | . . . . | . . . . |
tcdB_630erm        F V S L T F S I L E G I N A I I E V D L L S K S Y K L L I S G E L K I L M L N S N H I Q Q K I D Y I
tcdB_VPI10463      . . . . . . . . . . . . . . . . . . . . . . . . . . . . . . . . . . . . . . . . . . . . . . . . . .
tcdB_R20291        . . . . . . . . . . . . . . V . . . . . . . . . . . V . . . . . . . T . . A . . . S V . . . . . . .
Clustal Consensus  * * * * * * * * * * * * * * : * * * * * * * * * * * : * * * * * * *   * *   * * *   : * * * * * * *

                                          1460                    1470                    1480                    1490                    1500
                   . . . . | . . . . | . . . . | . . . . | . . . . | . . . . | . . . . | . . . . | . . . . | . . . . |
tcdB_630erm        G F N S E L Q K N I P Y S F V D S E G K E N G F I N G S T K E G L F V S E L P D V V L I S K V Y M D
tcdB_VPI10463      . . . . . . . . . . . . . . . . . . . . . . . . . . . . . . . . . . . . . . . . . . . . . . . . . .
tcdB_R20291        . L . . . . . . . . . . . . M . D K . . . . . . . . C . . . . . . . . . . . S . . . . . . . . . . .
Clustal Consensus  * : * * * * * * * * * * * * : * . : * * * * * * * *   * * * * * * * * * * * . * * * * * * * * * * *

                                          1510                    1520                    1530                    1540                    1550
                   . . . . | . . . . | . . . . | . . . . | . . . . | . . . . | . . . . | . . . . | . . . . | . . . . |
tcdB_630erm        D S K P S F G Y Y S N N L K D V K V I T K D N V N I L T G Y Y L K D D I K I S L S L T L Q D E K T I
tcdB_VPI10463      . . . . . . . . . . . . . . . . . . . . . . . . . . . . . . . . . . . . . . . . . . . . . . . . . .
tcdB_R20291        N . . . L . . . C . . D . . . . . . . . . . D . I . . . . . . . . . . . . . . . . F . I . . . N . .
Clustal Consensus  : * * *   * * *   * * : * * * * * * * * * * : *   * * * * * * * * * * * * * * * * : * : * * * : * *

                                          1560                    1570                    1580                    1590                    1600
                   . . . . | . . . . | . . . . | . . . . | . . . . | . . . . | . . . . | . . . . | . . . . | . . . . |
tcdB_630erm        K L N S V H L D E S G V A E I L K F M N R K G N T N T S D S L M S F L E S M N I K S I F V N F L Q S
tcdB_VPI10463      . . . . . . . . . . . . . . . . . . . . . . . . . . . . . . . . . . . . . . . . . . . . . . . . . .
tcdB_R20291        . . . G . Y . . . N . . . . . . . . . . K . . S . . . . . . . . . . . . . . . . . . . . I . S . . .
Clustal Consensus  * * * . * : * * * . * * * * * * * * * * . * * . * * * * * * * * * * * * * * * * * * * * : *   * * *

                                          1610                    1620                    1630                    1640                    1650
                   . . . . | . . . . | . . . . | . . . . | . . . . | . . . . | . . . . | . . . . | . . . . | . . . . |
tcdB_630erm        N I K F I L D A N F I I S G T T S I G Q F E F I C D E N D N I Q P Y F I K F N T L E T N Y T L Y V G
tcdB_VPI10463      . . . . . . . . . . . . . . . . . . . . . . . . . . . . . . . . . . . . . . . . . . . . . . . . . .
tcdB_R20291        . T . L . . . T . . . . . . . . . . . . . . . . . . K D N . . . . . . . . . . . . . . K . . . . . .
Clustal Consensus  *   * : * * * : * * * * * * * * * * * * * * * * * * : : : * * * * * * * * * * * * * * : * * * * * *

                                          1660                    1670                    1680                    1690                    1700
                   . . . . | . . . . | . . . . | . . . . | . . . . | . . . . | . . . . | . . . . | . . . . | . . . . |
tcdB_630erm        N R Q N M I V E P N Y D L D D S G D I S S T V I N F S Q K Y L Y G I D S C V N K V V I S P N I Y T D
tcdB_VPI10463      . . . . . . . . . . . . . . . . . . . . . . . . . . . . . . . . . . . . . . . . . . . . . . . . . .
tcdB_R20291        . . . . . . . . . . . . . . . . . . . . . . . . . . . . . . . . . . . . . . . . . I . . . . . . . .
Clustal Consensus  * * * * * * * * * * * * * * * * * * * * * * * * * * * * * * * * * * * * * * * * * : * * * * * * * *

                                          1710                    1720                    1730                    1740                    1750
                   . . . . | . . . . | . . . . | . . . . | . . . . | . . . . | . . . . | . . . . | . . . . | . . . . |
tcdB_630erm        E I N I T P V Y E T N N T Y P E V I V L D A N Y I N E K I N V N I N D L S I R Y V W S N D G N D F I
tcdB_VPI10463      . . . . . . . . . . . . . . . . . . . . . . . . . . . . . . . . . . . . . . . . . . . . . . . . . .
tcdB_R20291        . . . . . . I . . A . . . . . . . . . . . T . . . S . . . . I . . . . . . . . . . . . . . . S . . .
Clustal Consensus  * * * * * * : * * : * * * * * * * * * * * : * * * . * * * * : * * * * * * * * * * * * * * * . * * *

                                          1760                    1770                    1780                    1790                    1800
                   . . . . | . . . . | . . . . | . . . . | . . . . | . . . . | . . . . | . . . . | . . . . | . . . . |
tcdB_630erm        L M S T S E E N K V S Q V K I R F V N V F K D K T L A N K L S F N F S D K Q D V P V S E I I L S F T
tcdB_VPI10463      . . . . . . . . . . . . . . . . . . . . . . . . . . . . . . . . . . . . . . . . . . . . . . . . . .
tcdB_R20291        . . . . D . . . . . . . . . . . . T . . . . G N . I S D . I . . . . . . . . . . S I N K V . S T . .
Clustal Consensus  * * * * . * * * * * * * * * * * * . * * * * . : * : : : * : * * * * * * * * * * . : . : : *   : * *

                                          1810                    1820                    1830                    1840                    1850
                   . . . . | . . . . | . . . . | . . . . | . . . . | . . . . | . . . . | . . . . | . . . . | . . . . |
tcdB_630erm        P S Y Y E D G L I G Y D L G L V S L Y N E K F Y I N N F G M M V S G L I Y I N D S L Y Y F K P P V N
tcdB_VPI10463      . . . . . . . . . . . . . . . . . . . . . . . . . . . . . . . . . . . . . . . . . . . . . . . . . .
tcdB_R20291        . . . . V E . . L N . . . . . I . . . . . . . . . . . . . . . . . . . V . . . . . . . . . . . . I K
Clustal Consensus  * * * *   : * * : . * * * * * : * * * * * * * * * * * * * * * * * * * : * * * * * * * * * * * * : :

                                          1860                    1870                    1880                    1890                    1900
```

```
                    ....|....|....|....|....|....|....|....|....|....|
tcdB_630erm         NLITGFVTVGDDKYYFNPINGGAASIGETIIDDKNYYFNQSGVLQTGVFS
tcdB_VPI10463       ..................................................
tcdB_R20291         ......T.I.........D......V......G.....S.N.........
Clustal Consensus   ******.*:********* *****:******.*****.*.*********

                          1910      1920      1930      1940      1950
                    ....|....|....|....|....|....|....|....|....|....|
tcdB_630erm         TEDGFKYFAPANTLDENLEGEAIDFTGKLIIDENIYYFDDNYRGAVEWKE
tcdB_VPI10463       ..................................................
tcdB_R20291         ...........D.................T....V...G....A.I..QT
Clustal Consensus   ***********:***************** ****:***.****.*:**: 

                          1960      1970      1980      1990      2000
                    ....|....|....|....|....|....|....|....|....|....|
tcdB_630erm         LDGEMHYFSPETGKAFKGLNQIGDYKYYFNSDGVMQKGFVSINDNKHYFD
tcdB_VPI10463       ..................................................
tcdB_R20291         ..D.VY...TD..R..........D.F......I......N...KTF...
Clustal Consensus   **.*::***.:**.********** *:******:******.***:..***

                          2010      2020      2030      2040      2050
                    ....|....|....|....|....|....|....|....|....|....|
tcdB_630erm         DSGVMKVGYTEIDGKHFYFAENGEMQIGVFNTEDGFKYFAHHNEDLGNEE
tcdB_VPI10463       ..................................................
tcdB_R20291         ......S........Y................A.........D.......
Clustal Consensus   ****** ********:**************** *********:*******

                          2060      2070      2080      2090      2100
                    ....|....|....|....|....|....|....|....|....|....|
tcdB_630erm         GEEISYSGILNFNNKIYYFDDSFTAVVGWKDLEDGSKYYFDEDTAEAYIG
tcdB_VPI10463       ..................................................
tcdB_R20291         ..AL..............................................
Clustal Consensus   ** :**********************************************

                          2110      2120      2130      2140      2150
                    ....|....|....|....|....|....|....|....|....|....|
tcdB_630erm         LSLINDGQYYFNDDGIMQVGFVTINDKVFYFSDSGIIESGVQNIDDNYFY
tcdB_VPI10463       ..................................................
tcdB_R20291         I.I....K.....S....I......NE.........V...M.........
Clustal Consensus   :*:****:*****.****:******::*********:***:*********

                          2160      2170      2180      2190      2200
                    ....|....|....|....|....|....|....|....|....|....|
tcdB_630erm         IDDNGIVQIGVFDTSDGYKYFAPANTVNDNIYGQAVEYSGLVRVGEDVYY
tcdB_VPI10463       ..................................................
tcdB_R20291         ..E..L............................................
Clustal Consensus   **:**:********************************************

                          2210      2220      2230      2240      2250
                    ....|....|....|....|....|....|....|....|....|....|
tcdB_630erm         FGETYTIETGWIYDMENESDKYYFNPETKKACKGINLIDDIKYYFDEKGI
tcdB_VPI10463       ..................................................
tcdB_R20291         ........................D......Y....V..........N..
Clustal Consensus   ************************:****** ****:**********:**

                          2260      2270      2280      2290      2300
                    ....|....|....|....|....|....|....|....|....|....|
tcdB_630erm         MRTGLISFENNNYYFNENGEMQFGYINIEDKMFYFGEDGVMQIGVFNTPD
tcdB_VPI10463       ..................................................
tcdB_R20291         ......T..D.H.....D.I..Y..L.....T...S...I..........
Clustal Consensus   ******:**:*:*****:* **:**:***** ***.***:**********

                          2310      2320      2330      2340      2350
                    ....|....|....|....|....|....|....|....|....|....|
tcdB_630erm         GFKYFAHQNTLDENFEGESINYTGWLDLDEKRYYFTDEYIAATGSVIIDG
```

```
tcdB_VPI10463      . . . . . . . . . . . . . . . . . . . . . . . . . . . . . . . . . . . . . . . . . . . . . . . . . .
tcdB_R20291        . . . . . . . . . . . . . . . . . . . . . . . . . . . . . . . . . . . . . . . . . . . . . . . . . .
Clustal Consensus  * * * * * * * * * * * * * * * * * * * * * * * * * * * * * * * * * * * * * * * * * * * * * * * * * *
```

```
                                   2360
                   . . . . | . . . . . | . . . . . | .
tcdB_630erm        E E Y Y F D P D T A Q L V I S E
tcdB_VPI10463      . . . . . . . . . . . . . . . .
tcdB_R20291        . . . . . . . . . . . . . . . .
Clustal Consensus  * * * * * * * * * * * * * * * *
```
