# Supplementary material for: A Streamlined Method to Obtain Biologically Active TcdA and TcdB Toxins from Clostridioides difficile
Source: Toxins (Basel). 2024 Jan 11;16(1):38. doi: 10.3390/toxins16010038 (PMC10821508; doi:10.3390/toxins16010038)
Supplement: Supplementary file 1 [file toxins-16-00038-s001.zip › File S2.pdf]

toxins

Supplementary Materials

A Streamlined Method to Obtain Biologically Active TcdA and TcdB Toxins from *Clostridioides difficile*

Diane Sapa [1,†], Anaïs Brosse [1,*,†], Héloïse Coullon [1], Gauthier Péan de Ponfilly [1,2], Thomas Candela [1,‡] and Alban Le Monnier [1,2,‡]

1 Micalis Institute, Université Paris-Saclay, INRAE, AgroParisTech, 78350 Jouy-en-Josas, France
2 Service de Microbiologie Clinique, GH Paris Saint-Joseph, 75674 Paris, France
* Correspondence: anais.brosse@universite-paris-saclay.fr
† Diane Sapa and Anaïs Brosse contributed equally to this work and share first authorship.
‡ Thomas Candela and Alban Le Monnier also contributed equally to this work and share last authorship.

Citation: Sapa, D.; Brosse, A.; Coullon, H.; Péan de Ponfilly, G.; Candela, T.; Le Monnier, A. A Streamlined Method to Obtain Biologically Active TcdA and TcdB Toxins from *Clostridioides difficile*. *Toxins* **2024**, *16*, 38. https://doi.org/10.3390/toxins16010038

Received: 25 September 2023
Revised: 15 December 2023
Accepted: 30 December 2023
Published: 11 January 2024

Copyright: © 2024 by the authors. Licensee MDPI, Basel, Switzerland. This article is an open access article distributed under the terms and conditions of the Creative Commons Attribution (CC BY) license (https://creativecommons.org/licenses/by/4.0/).

Supplementary Materials and Methods:

DNase assay: To assess whether DNA contamination could be an issue, DNA quantification was done before and after incubation with 70 µg.mL^{-1} DNase I (CAS: 9003-98-9, Sigma Aldrich, MO, USA)for 2 hours at 37°C. In addition, positive control was included by spiking the mixture with 1 µL of a DNA sample at 170.47 ng.µL^{-1}.

Western blot: Purified recombinant toxins rTcdA and rTcdB were separated on 12% Mini-PROTEAN TGX Stain-Free Gels (Bio-Rad, CA, USA) and then transferred to a nitrocellulose membrane using Trans-Blot Turbo Transfer System (Bio-Rad, CA, USA) according to the manufacturer's instructions. Purified recombinant toxins rTcdA and rTcdB were detected using anti-His primary antibody (#2365S, Cell Signaling, MA, USA) diluted at 1:1000 and anti-rabbit horseradish peroxidase (HRP)-linked secondary antibody diluted at 1:20000 (GeneTex GTX213110-01, CA, USA). Antibody binding was revealed with SuperSignal™ West Pico PLUS Chemiluminescent Substrate (reference: 34580, Thermo Scientific, MA, USA) and revelation was performed on Fusion FX7 Imaging System (Vilber Lourmat, Eberhardzell, Germany).

Toxin expression assay: *C. difficile* strains AD3 and AD4 were cultured in 15 mL of BHI medium and allowed to grow overnight in an anaerobic chamber at 37°C and toxin expression was induced by the addition of ATc at 0.1 µg.mL^{-1} or 0.5 µg.mL-1 to 5 mL bacteria culture. Bacteria were centrifuged and culture supernatants were precipitated with 45 g of ammonium sulfate for 100 mL of bacterial culture followed by shaking at room temperature for 5 min. Precipitates were centrifugated (12200 g) for 10 min at 4 °C, and the pellet was resuspended in 100 µL of Laemmli sample buffer. For analysis, supernatant before (S) and after precipitation (CS) were loaded into a sodium dodecyl-sulfate polyacrylamide gel electrophoresis (SDS-PAGE) gel using 6% stacking gel and 10% separation gel followed by a standard Coomassie staining.

Disclaimer/Publisher's Note: The statements, opinions and data contained in all publications are solely those of the individual author(s) and contributor(s) and not of MDPI and/or the editor(s). MDPI and/or the editor(s) disclaim responsibility for any injury to people or property resulting from any ideas, methods, instructions or products referred to in the content.
