# Supplementary material for: A Streamlined Method to Obtain Biologically Active TcdA and TcdB Toxins from Clostridioides difficile
Source: Toxins (Basel). 2024 Jan 11;16(1):38. doi: 10.3390/toxins16010038 (PMC10821508; doi:10.3390/toxins16010038)
Supplement: Supplementary file 1 [file toxins-16-00038-s001.zip › toxins-2657655 supplementary figures and tables.pdf]

toxins

MDPI

Supplementary Materials: A Streamlined Method to Obtain Biologically Active TcdA and TcdB Toxins from *Clostridioides difficile*

Diane Sapa, Anaïs Brosse, Héloïse Coullon, Gauthier Péan de Ponfilly, Thomas Candela and Alban Le Monnier

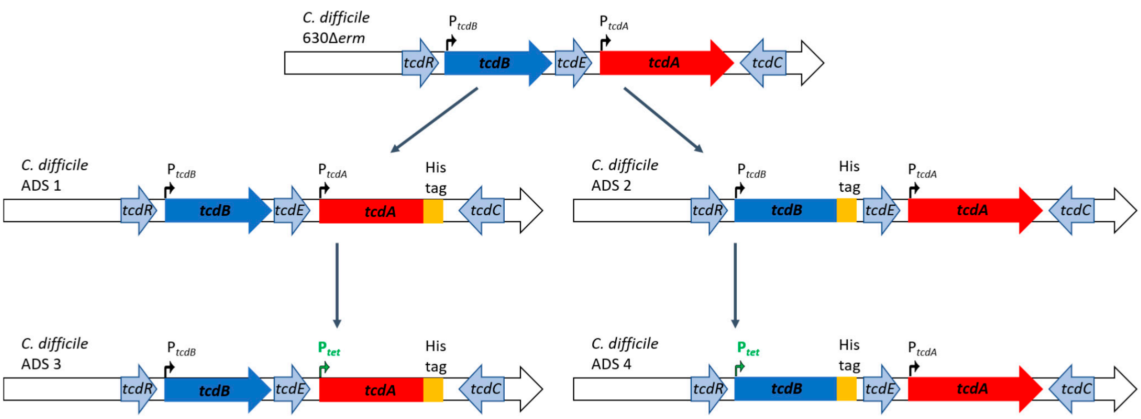

Figure S1. Strain construction for the production of the recombinant toxins rTcdA and rTcdB of *C. difficile*. The strains were obtained in two steps using the allele exchange method. Strains ADS1 and ADS2 were obtained by adding a His-tag at the 3′ end of the *tcdA* or *tcdB* genes of the *C. difficile* strain 630Δ*erm*. Strains ADS3 and ADS4 were obtained by replacing the respective promoter of *tcdA* and *tcdB* by the promoter Ptet inducible to anhydro-tetracycline (ATc). AD3 and ADS4 were used for the purification of recombinant toxin A (rTcdA) and B (rTcdB).

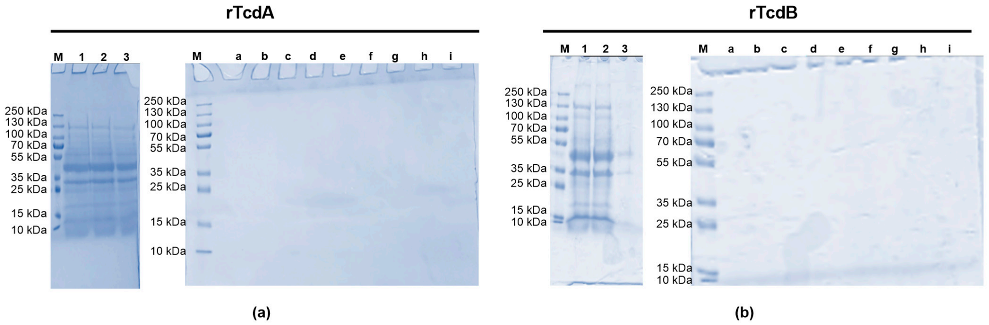

Figure S2. Purification of recombinant His-tagged toxins by Ni-NTA affinity chromatography under denaturing conditions from the genetic construction without the Ptet promoter. Toxins were visualized by Coomassie staining for (**A**) recombinant toxin A (rTcdA); (**B**) recombinant toxin B (rTcdB). M: PageRuler Plus marker (ThermoFisher) / 1: raw protein samples / 2: flow through / 3: wash. Imidazole eluted fractions: a: 10 mM / b: 20 mM / c: 30 mM / d: 40 mM / e: 60 mM / f: 80 mM / g: 100 mM / h: 200 mM / i: 1 M.

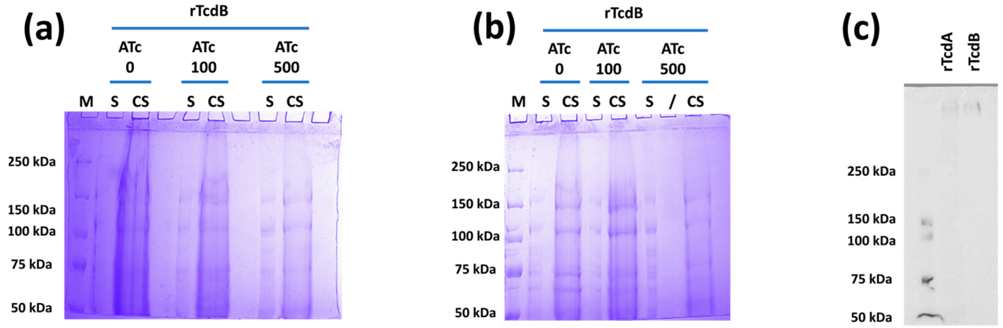

(d)

DNA quantification (ng/µL)	rTcdA	rTcdB
Recombinant protein	12.5	18.6
DNAse-treated recombinant protein	9.8	9.7
Recombinant protein + DNA spike in	23	38.3

Figure S3. Validation of the recombinant protein extraction method. Absence of recombinant proteins in culture supernatant was confirmed for rTcdA and rTcdB (panels **a** and **b** respectively). Specificity of extracted recombinant proteins was confirmed by His-tag directed western-blot (panel **c**). DNA contamination in the extracted protein samples was estimated by DNA quantification using the nanodrop before and after DNAse treatment, using spiked-in DNA as a control (panel **d**). For experimental details, see Supplemental File S2. M: Page Ruler Plus ladder (ThermoFisher), S: raw culture supernatant, CS: Concentrated supernatant after protein precipitation.

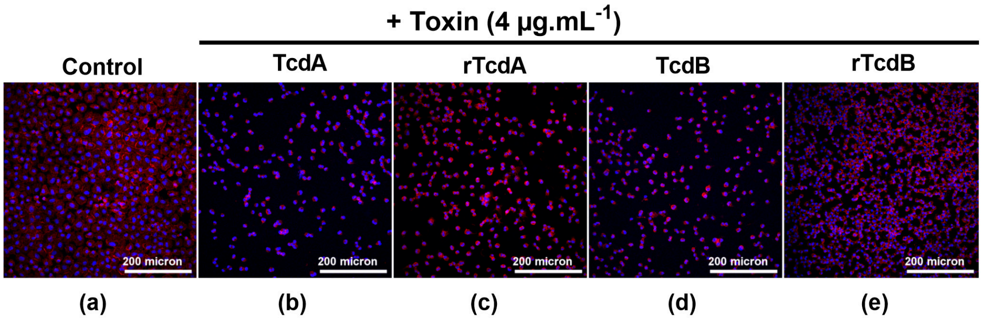

Figure S4. Disruption of cytoskeleton on Vero cells with 20x objective. Cells in 24 wells plate were in the absence of toxin (panel a = control) or in the presence of 4 µg.ml^{-1} of either native or recombinant toxins **A** (panel b = native / panel c = recombinant) and **B** (panel d = native / panel e = recombinant). Cells were incubated for 18 hours, fixed, and stained with Rhodamine Phalloidin (actin staining in red) and DAPI (nuclei staining in blue) then observed under confocal microscopy with 20× objective to quantify the effect of toxins on the cells.

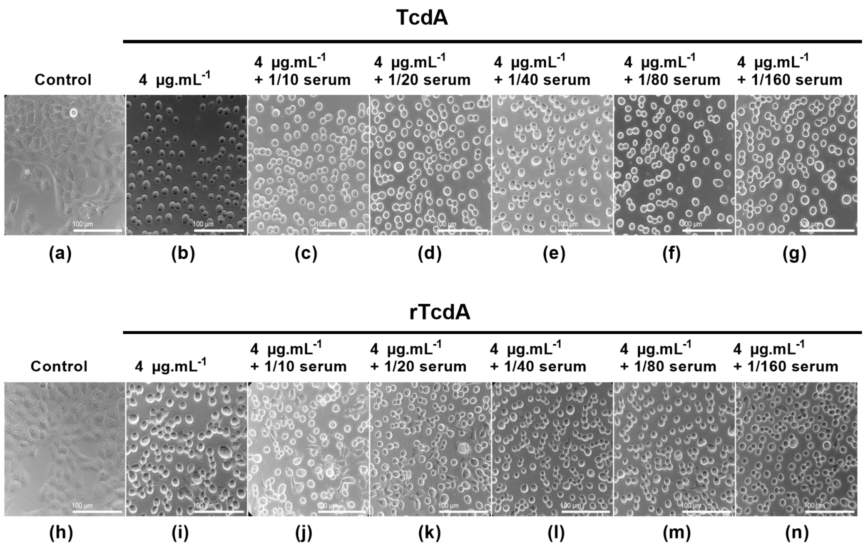

Figure S5. Serum without anti-TcdA neutralizing IgG does not neutralize TcdA toxins activity. Toxins were preincubated with several sera from a CDI-convalescent patient using dilutions ranging from 1/10 (panels **c** and **j**) to 1/160 (panels **g** and **n**) before addition to Vero cell monolayers. Native VPI 10463 TcdA and recombinant TcdA were compared (panels **b**-**g** and **i**-**n** respectively). Cells incubated without toxins (panels **a** and **h**) were included as a negative control. Cells incubated with non-neutralized toxins were included as a positive control (panels **b** and **i**). Morphological changes were observed under phase contrast microscopy after 18 h at 37 °C.

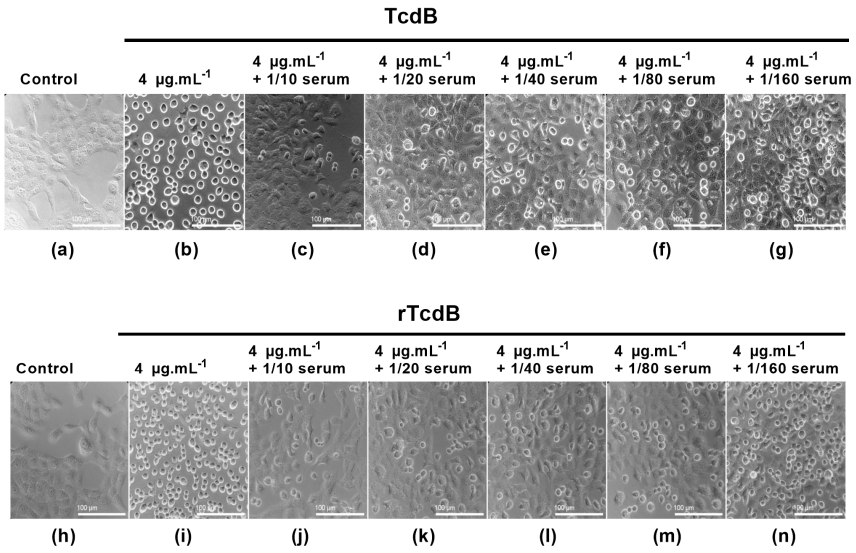

Figure S6. Serum with anti-TcdB neutralizing IgG neutralizes both native and recombinant TcdB activity. Toxins were preincubated with several sera from a CDI-convalescent patient using dilutions ranging from 1/10 (panels **c** and **j**) to 1/160 (panels **g** and **n**) before addition to Vero cell monolayers. Native VPI 10463 TcdB and recombinant TcdB were compared (panels **b**-**g** and **i**-**n** respectively). Cells incubated without toxins (panels **a** and **h**) were included as a negative control. Cells incubated with non-neutralized toxins were included as a positive control (panels **b** and **i**). Morphological changes were observed under phase contrast microscopy after 18 h at 37 °C.

Table S1. List of bacterial strains used in this study.

Strains	Characteristics	Reference(s)/source
TG1	*E.coli* SupE *hsd*Δ5 thiΔ(lac-proAB) *F'*[*tra36 pro AB*+*lacI*q *lac*ZΔM15]	[59]
HB101*tra*	*E. coli,* F-Δ (gpt-proA) 62 LEU B6 gln V44 ara-14 gal-K2 lacY1 delta (mcrC-mrr) rps L20 (srtr) xyl-5 mLt-1 recA13, pRK24 (transfer function)	Laboratory
630Δ*erm*	*C. difficile,* Wild-type strain sensible to erythromycin	Laboratory
AD1	*C. difficile,* 630Δ*erm* with 6xhis-tag in the 3′ end of *tcdA*	This study
AD2	*C. difficile,* 630Δ*erm* with 6xhis-tag in the 3′end of *tcdB*	This study
AD3	*C. difficile* strain AD1 with *tcdA* native promoter replaced with P_{tet} promoter (630Δ*erm* P_{tet}-TcdA_his tag)	This study
AD4	*C. difficile* strain AD2 with *tcdB* native promoter replaced with P_{tet} promoter (630Δ*erm* P_{tet}-TcdB_his tag)	This study

Table S2. List of primers used in this study.

Name	Sequence 5′ – 3′	Utilization
2836	ctgcacccgggGAGACCGGAAAAGATCCGGGG-GATCGATCCTCTAG	Primer for amplification of the spectinomycin cassette
2837	catgtcgcatgcGAGACCTTAGCCTAATTGAGA-GAAGTTTCTATAG	Primer for amplification of the spectinomycin cassette
2854	GGCTACGGTCTCCCCGGGgcctcaactggttata-caagtattaatgg	Primer for the amplification of PCR fragment 1 for pADS1 construction
2851	GGCTACGGTCTCCGGCAttaG-tGGTGATGGTGATGGTGATGgccatatatcccaggggct tttactcc	Primer for the amplification of PCR fragment 1 for pADS1 construction
2852	GGCTACGGTCTCCTGCCaatatatgtttga-taaaaaaattattcctgtg	Primer for for amplification of PCR fragment 2 for pADS1 construction
2853	GGCTACGGTCTCGCATGCggtaacgaatttag-taatgaaggaaaagg	Primer for amplification of PCR fragment 2 for pADS1 construction
2858	GGCTACGGTCTCCCCGGGgctgaaaacgga-gaaatgcaaataggag	Primer for amplification of PCR fragment 1 for pADS2 construction
2859	GGCTACGGTCTCCGGCAttaG-tGGTGATGGTGATGGTGATGttcactaatcactaatt-gagctgtatcaggatc	Primer for amplification of PCR fragment 1 for pADS2 construction
2860	GGCTACGGTCTCCTGCCataaaaa-tatgttaaatatatcctcttatacttaa	Primer for amplification of PCR fragment 2 for pADS2 construction
2861	GGCTACGGTCTCGCATGCgtccttcatcacgtatttttt-gtgtttttg	Primer for amplification of PCR fragment 2 for pADS2 construction
3046	gatgagaagggcataatgggctacggtctctttgc	Forward primer for CO1 fragment amplification for pADS3 construction
3047	ttacttttcatccttagcattcggctacggtctcttcttatttttatgctagc	Reverse primer for CO1 fragment amplification for pADS3 construction
3048	taagaagcctgcatttgcagggctacggtctc	Forward primer for P_{tet} fragment amplification for pADS3 construction
3049	aaagacataaaattttctcctttactgcaggggctacggtctcg	Reverse primer for P_{tet} fragment amplification for pADS3 construction
3050	ctttaatatctaaagaagagttaataaaacggctacggtctcg	Forward primer for CO2 fragment amplification for pADS3 construction
3051	ctgccgcctctaattttatcatttccggctacggtctc	Reverse primer for CO2 fragment amplification for pADS3 construction

3052	ttatgtaattgttacttgaaaattgatcggctacggtctcattgc	Forward primer for CO1 fragment amplification for pADS4 construction
3053	ttacaagttaaaataattttcatagtcttttttatttttttaaatgggctac-ggtctcgtcttatttttatgctagc	Reverse primer for CO1 fragment amplification for pADS4 construction
3054	aagaagcctgcatttgcagggctacggtctcg	Forward primer for P_{tet} fragment amplification for pADS4 construction
3055	aaaattttctcctttactgcaggggctacggtctcgactaaactcat	Reverse primer for P_{tet} fragment amplification for pADS4 construction
3056	tagttaatagaaaacagttagaaaaaaatgggctacggtctcg	Forward primer for CO2 fragment amplification for pADS4 construction
3057	tgttctgaggtatattctggggctacggtctcttgcc	Reverse primer for CO2 fragment amplification for pADS4 construction
3064	tatacaattgagactggatggat	Forward primer outside CO1 fragment to verify P_{tet} promoter's insertion in front of *tcdA* in *C. difficile*
3065	aatatctcagatttttcactttt	Reverse primer outside CO2 to verify P_{tet} promoter's insertion in front of *tcdA* in *C. difficile*
3066	gtcttgaaaatatttagtttgaa	Forward primer outside CO1 fragment to verify P_{tet} promoter's insertion in front of *tcdB* in *C. difficile*
3067	attttaacttctagtggtgatgc	Reverse primer outside CO2 to verify P_{tet} promoter's insertion in front of *tcdB* in *C. difficile*
3068	ctgtttttctgtaggccgtgt	Forward primer inside P_{tet} promoter to verify P_{tet}'s insertion in front of *tcdA* and *tcdB* in *C. difficile*
3069	acacggcctacagaaaaacag	Reverse primer inside P_{tet} promoter to verify P_{tet}'s insertion in front of *tcdA* and *tcdB* in *C. difficile*
3070	gctgttaataaggctaaaaatagagca	Forward primer inside CO1 fragment to verify P_{tet} promoter's insertion in front of *tcdB* in *C. difficile*
3071	aaggctttatttctaccagatttt	Reverse primer inside CO2 to verify P_{tet} promoter's insertion in front of *tcdB* in *C. difficile*
3072	gaaagtgtaagtatattaaaaaatat	Forward primer inside CO1 fragment to verify P_{tet} promoter's insertion in front of *tcdA* in *C. difficile*
3073	cattattgtttgtagttaacttat	Reverse primer inside CO2 to verify P_{tet} promoter's insertion in front of *tcdA* in *C. difficile*
3143	tcaaagcggcaactaagagt	Forward primer outside CO1 fragment in pJV7 vector to characterize the first Crossing-over between pADS3 and gDNA in AD3 and between pADS4 and gDNA in AD4
3144	tgtaaaacgacggccagt	Reverse primer outside CO2 fragment in pJV7 vector to characterize the first Crossing-over between pADS3 and gDNA in AD3 and between pADS4 and gDNA in AD4
3145	tggcatctgctccctcgttttcat	Reverse primer inside *tcdA* native promoter in front of CO2 fragment to characterize the first Crossing-over between pADS3 and gDNAin AD3
3146	aggagtgtataagatttattttc	Forward primer inside *tcdA* native promoter in front of CO2 fragment to characterize the first Crossing-over between pADS3 and gDNA in AD3
3147	aagacactcagttgattaatttgc	Reverse primer inside *tcdB* native promoter in front of CO2 fragment to characterize the first Crossing-over between pADS4 and gDNA in AD4
3148 DS30	gcaaattaatcaactgagtgtctt	Forward primer inside *tcdB* native promoter in front of CO2 fragment to characterize the first Crossing-over between pADS4 and gDNA in AD4

Table S3. List of Plasmids used in this study.

Name	Characteristics	Reference (s)/Source
pAT28	Spec[r] ori pUC ori pAmβ1 (resistance spc)	[43]
pTC129	pBlunt+ spc (BsaI) (resistance Kn+spc)	This study
pTC130	pMSR BamH1/XhoI +pTC129 BamH1/xhoI (resistance Cm+spc)	This study
pADS1	pTC130 with 6xhis - tag for allele exchange in *C. difficile* 630Δ*erm* to generate strain AD1	This study
pADS2	pTC130 with 6xhis - tag for allele exchange in *C. difficile* 630Δ*erm* to generate strain AD2	This study
pRPF185	pRPF177 with fdx transcriptional terminator added after *tetR, gusA*	[46]
pJV7	pMSR spc (BsaI) (resistance Cm+spc)	[47]
pADS3	P_{tet} promoter and homologous regions cloned in pJV7 vector for allele exchange upstream of *tcdA* in *C. difficile* strain AD1 to generate strain AD3	This study
pADS4	P_{tet} promoter and homologous regions cloned in pJV7 vector for allele exchange upstream of *tcdB* in *C. difficile* strain AD2 to generate strain AD4	This study

Table S4. List of Genome accession numbers used for Figure 1.

Genome Name / Sample Name	Genome ID
Clostridioides difficile E12	CAMZ00000000
Clostridioides difficile E28	CAMX00000000
Clostridioides difficile T11	CAML00000000
Clostridioides difficile NAP08	ADNX00000000
Clostridioides difficile NAP07	ADVM00000000
Clostridioides difficile QCD-66c26	CM000441
Clostridioides difficile 630 delta erm	LN614756
Clostridioides difficile T19	CANA00000000
Clostridioides difficile T61	CAND00000000
Clostridioides difficile QCD-97b34	CM000657
Clostridioides difficile T17	CAMT00000000
Clostridioides difficile E15	CAMM00000000
Paeniclostridium sordellii ATCC 9714	APWR00000000
Clostridioides difficile T6	CAMR00000000
Clostridioides difficile E24	CAMP00000000
Clostridioides difficile 630	AM180355, AM180356
Clostridioides difficile E14	CAMS00000000
Clostridioides difficile QCD-23m63	CM000660
Clostridioides difficile CD15	FUUU00000000
Clostridioides difficile T15	CAMK00000000
Clostridioides difficile CIP 107932	CM000659
Clostridioides difficile E25	CAMJ00000000
Clostridioides difficile E16	CAMH00000000
Clostridioides difficile QCD-32g58	CM000287
Clostridioides difficile T42	CAMQ00000000
Clostridioides difficile QCD-63q42	CM000637
Clostridioides difficile E23	CAMY00000000
Clostridioides difficile QCD-37x79	CM000658
Clostridioides difficile R20291	FN545816
Clostridioides difficile T22	CAMI00000000
Clostridioides difficile T10	CANB00000000

Clostridioides difficile E1	CAMD00000000
Clostridioides difficile QCD-76w55	CM000661
Clostridioides difficile T5	CAMB00000000
Clostridioides difficile T20	CAMC00000000
Clostridioides difficile E7	CAMV00000000
Clostridioides difficile T23	CAMN00000000
Clostridioides difficile T3	CAMW00000000
Clostridioides difficile CD196	FN538970
Clostridioides difficile A685	https://mage.genoscope.cns.fr/microscope/search/export-Form.php?O_id=1276
